# Stepwise Synthesis of Carboxyalumoxanes through Well-Defined Organoaluminum and Organogallium Carboxylatohydroxides Obtained by Controlled Hydrolysis

**DOI:** 10.1021/acs.inorgchem.5c00367

**Published:** 2025-05-01

**Authors:** Wanda Ziemkowska, Vadim Szejko, Bernadeta Prus, Paweł Socha, Michał K. Cyrański, Agnieszka Jastrzębska, Iwona Justyniak

**Affiliations:** † Warsaw University of Technology, Faculty of Chemistry, Noakowskiego 3, 00-664 Warsaw, Poland; ‡ Department of Chemistry, University of Warsaw, Pasteura 1, 02-093 Warsaw, Poland; § Warsaw University of Technology, Faculty of Mechatronics, św. Andrzeja Boboli 8, 02-526 Warsaw, Poland; ∥ Institute of Physical Chemistry Polish Academy of Sciences, Kasprzaka 44/52, 01-224 Warsaw, Poland

## Abstract

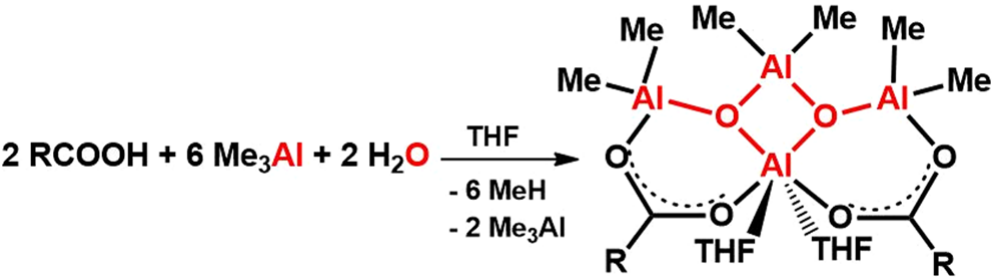

Alumoxanes are compounds of composition (R_2_AlOAlR_2_)*_n_* and (RAlO)*_n_* that are traditionally obtained by controlled hydrolysis of aluminum trialkyls, although nonhydrolytic approaches to alumoxanes are also used. To reduce the reactivity of alumoxanes in contact with air, in the 1990s, researchers attempted to replace alkyl groups with other substituents, leading to the synthesis of carboxyalumoxanes with bulky alkyl groups. In this paper, we report on the study of carboxy methylalumoxane synthesis and structural characterization. We present a two-step synthesis of carboxyalumoxane containing the (*t*-Bu_2_Al)_2_OAlMe_2_ alumoxane unit. The method involves the synthesis of carboxylatoaluminum hydroxides by the reaction of *t*-Bu_3_Al with carboxylic acid and water with a molar ratio of reagents of 2:1:1 in the first step, followed by the reaction with Me_3_Al. On the other hand, the one-step reaction of Me_3_Al with carboxylic acid and water in a molar ratio of 3:1:1 leads to the formation of a carboxy methylalumoxane incorporating a neutral Me_6_Al_4_O_2_ scaffold trapped by two carboxylate units. The reaction of carboxylatogallium hydroxides with Me_3_Al occurred with the exchange of *t*-Bu_2_Ga groups for Me_2_Al, leading to the formation of carboxyalumoxanes with a Me_6_Al_4_O_2_ scaffold. The molecular and crystal structures of the compounds were determined via single-crystal X-ray diffraction (SCXRD) crystallography.

## Abstract 2

Alumoxanes are compounds of composition (R_2_AlOAlR_2_)*_n_* and (RAlO)*_n_* that are traditionally obtained by controlled hydrolysis of aluminum trialkyls, although non-hydrolytic approaches to alumoxanes are also use.

## 1 Introduction

**Scheme 1:**
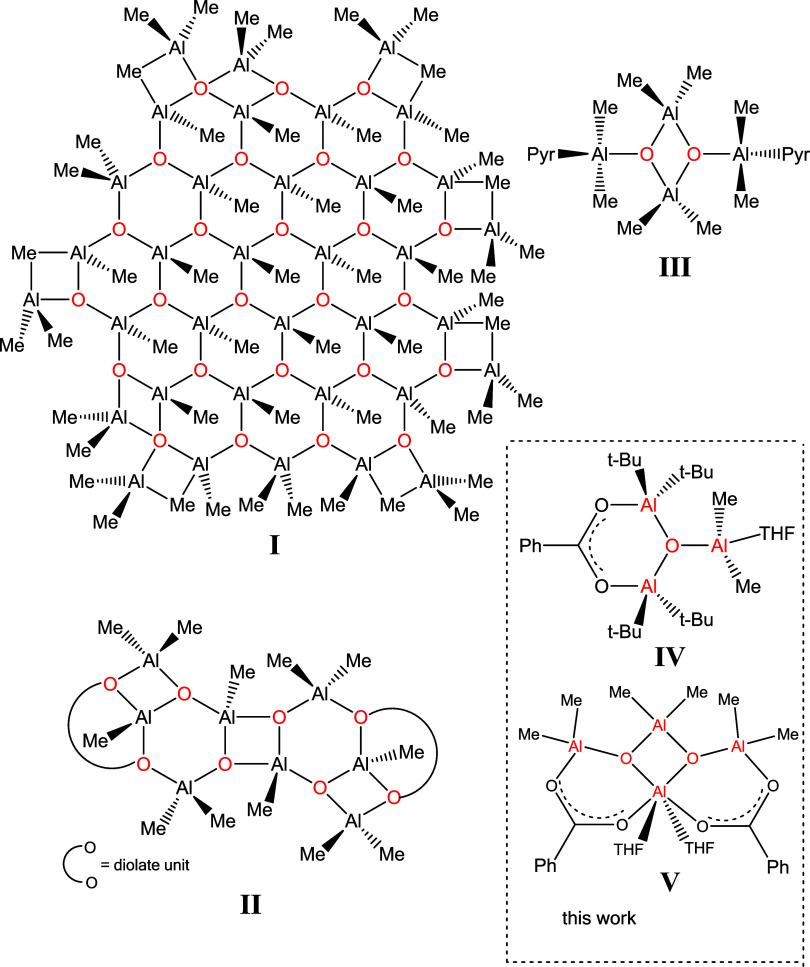
Selected Structurally Characterized Compounds Bearing Methylalumoxane Motifs; Me = Methyl, Ph = Phenyl, *t*-Bu = *tert*-Butyl, pyr = Pyridine, and THF = Tetrahydrofuran

For more than 50 years, the hydrolysis of group 13 organometallic compounds, and in particular aluminum derivatives, has been intensively studied due to the numerous applications of the hydrolysis products in catalysis and materials engineering. During the controlled hydrolysis of aluminum trialkyls, in addition to various alkylaluminum hydroxides, oligomeric compounds are formed with the composition of (R_2_AlOAlR_2_)*_n_* and (RAlO)*_n_* depending on the molar ratio of the reactants. They belong to the general class of compounds called alumoxanes, characterized by the presence of at least one oxo bridging group between two metallic centers.^1−10^ Many alumoxanes, along with other products of alkylaluminum hydrolysis, are used in organic synthesis and as catalysts for the polymerization of olefins, propylene oxides and ketones, and intermediates in the synthesis of nanoalumina.^11−17^ From the point of view of industrial application and in organic syntheses, the most important compound in this class is oligomeric methylalumoxane (MAO), which acts as a cocatalyst in metallocene-based Ziegler–Natta catalytic systems MAO and is a large-volume industrial product.^18,19^ Regardless of the numerous approaches to the structural characterization of MAO involving different synthesis routes and postsynthetic protocols, they were unsuccessful due to the complexity of the product mixture of Me_3_Al hydrolysis,^20−27^ having only recently, in 2024, been successful when discrete two-dimensional sheet cluster [Al_33_O_26_Me_47_][AlMe_3_]_2_ was revealed using single-crystal X-ray diffraction (SCXRD) analysis (Scheme 1, **I**).^28^ Over the years, due to the impossibility of experimentally determining the structures of MAO, the emphasis was directed toward the theoretical explanation of their nature and a number of computed structures were proposed, such as linear and cyclic polymers, sheet-like particles and cages.^23−34^ Conversely, the use of bulky substituents on the aluminum center has allowed the characterization of alumoxanes such as *t*-Bu_7_Al_5_O_3_(OH)_2_, *t*-Bu_8_Al_6_O_4_(OH)_2_, and (*t*-BuAlO)_8_ isolated by Barron et al., while Roesky isolated and determined the crystal structure of numerous hydrolysis products of aluminum compounds with bulky dimethyl tris(trimethylsilyl) groups.^10,35^

Due to the low stability of alumoxanes on air/moisture exposure and the consequent limited ability of their application, there were efforts to replace active alkyl groups with other, less reactive substituents. In the 1990s, interest attracted carboxyalumoxanes, where some alkyl groups were replaced by carboxylate anions, increasing their resistance to water. The carboxyalumoxanes were prepared by Barron, reacting boehmite with a series of aliphatic carboxylic acids in boiling solvents under anhydrous conditions.^36^ Florjańczyk used tri-*iso-*propoxy aluminum instead of boehmite in reactions with carboxylic acids. However, instead of the expected carboxyalumoxanes, high molecular weight coordination polymers without Al–O–Al alumoxane motif were obtained.^37,38^ Despite recognizable interest in carboxyalumoxanes, only three examples of structurally characterized carboxyalumoxanes are known to date, featuring an oligomeric cage structure upon the presence of bulky substituents on the aluminum centers. Two of the structurally characterized compounds [(*t*-Bu)_5_Al_5_(μ_3_-O)_2_(μ_3_–OH)_2_(μ–OH)_2_(μ-O_2_CPh)_2_] and [(*t*-Bu)_6_Al_6_(μ_3_-O)_4_(μ–OH)_2_(μ-O_2_CCCl_3_)_2_] were obtained by reacting of the cage alumoxane [*t*-BuAl(μ_3_-O)]_6_ with benzoic and trichloroacetic acids, respectively.^39,40^ The last structurally characterized organoalumoxane carboxylate was synthesized in 2011 in the reaction of one equivalent of (Me_3_Si)_3_CAlMe_2_ with one equivalent of 3,5-di*tert*-butyl salicylic acid monohydrate.^41^ As in the case of classical alumoxanes, carboxyalumoxanes containing Me or Et groups at the aluminum center are challenging to characterize in terms of crystallography, and their structures have only been postulated employing spectroscopic techniques.^7,23^

Very recently, Pietrzykowski et al. reported a series of aluminum compounds containing methylalumoxane aggregate, [Me_10_Al_6_O_4_], flanked by methylaluminum diolate units formed through nonhydrolytic alkylation of dicarboxylic acids (Scheme 1, **II**),^42^ spearheading the earlier efforts on nonhydrolytic approaches to alumoxanes.^23,43−46^

Although tetraalkylalumoxanes R_2_AlOAlR_2_ (where R = Et, i-Bu, *t*-Bu) have been known for years, the crystal structure of tetramethylalumoxane was reported only in 2024 by Lewiński et al. applying a route to tetramethylalumoxane by the controlled hydrolysis of Me_3_Al in the presence of pyridine as strong coordinating ligand (Scheme 1, **III**).^5,8,10,47^

The introduction of carboxyl groups into the structure of alumoxanes allows obtaining a new group of compounds for specific applications such as hybrid fillers for polymers, components of polymer networks, precursors of ceramic nanomaterials, and potential catalysts for olefin polymerization. Stemming from our extensive study on aluminum carboxylates and methylalumoxanes,^42,47^ herein, we report on the stepwise synthesis of carboxy methylalumoxanes through well-defined organoaluminum and organogallium carboxylatohydroxides. We present the synthesis and structural characterization of carboxy methylalumoxanes incorporating a neutral methylalumoxane Me_6_Al_4_O_2_ scaffold trapped by two carboxylate units (Scheme 1, structures **IV** and **V**). Our findings provide deeper insight into the fundamental reactivity of aluminum- and gallium hydroxides with trimethylaluminum, paving the way for further developments in alumoxane chemistry.

## 2 Experimental Section

### 2.1 Materials and Instrumentation

**Figure 1:**
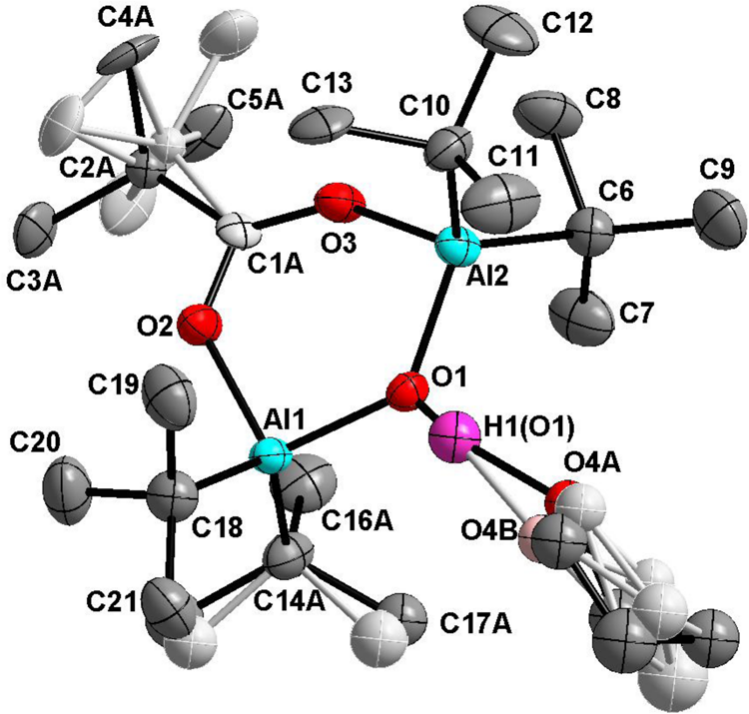
Thermal ellipsoid plot (50% probability) of compound **3**. Hydrogen atoms (besides H1(O1) atom) have been omitted for the sake of clarity. Positions of THF and two *t*-Bu group atoms are disordered. Selected bonds and distances (Å) and angles (°): Al(1)–O(1) 1.825(3), Al(1)–O(2) 1.849(3), Al(1)–C(18) 1.979(8), Al(2)–O(1) 1.830(3), Al(2)–O(3) 1.848(3), Al(2)–C(6) 1.930(8), Al(2)–C(10) 1.981(5), C(1A)-C(2A) 1.52(1), O(1)–Al(1)–O(2) 98.0(1), O(1)–Al(1)–C(18) 114.3(3), O(2)–Al(1)–C(18) 101.3(2), O(1)–Al(1)–C(20) 146.6(2), O(2)–Al(1)–C(20) 78.1(2), C(18)–Al(1)–C(20) 37.4(3), O(1)–Al(2)–O(3) 97.6(1) O(1)–Al(2)–C(6) 116.9(3), O(3)–Al(2)–C(6) 102.5(2), O(1)–Al(2)–C(10) 111.2(2), O(3)–Al(2)–C(10) 106.9(2), C(6)–Al(2)–C(10) 118.3(3), O(1)–Al(2)–C(8) 147.1(2), O(3)–Al(2)–C(8) 75.5(2), C(6)–Al(2)–C(8) 38.3(3), C(10)–Al(2)–C(8) 101.5(2), Al(1)–O(1)–Al(2) 127.9(1).

**Figure 2:**
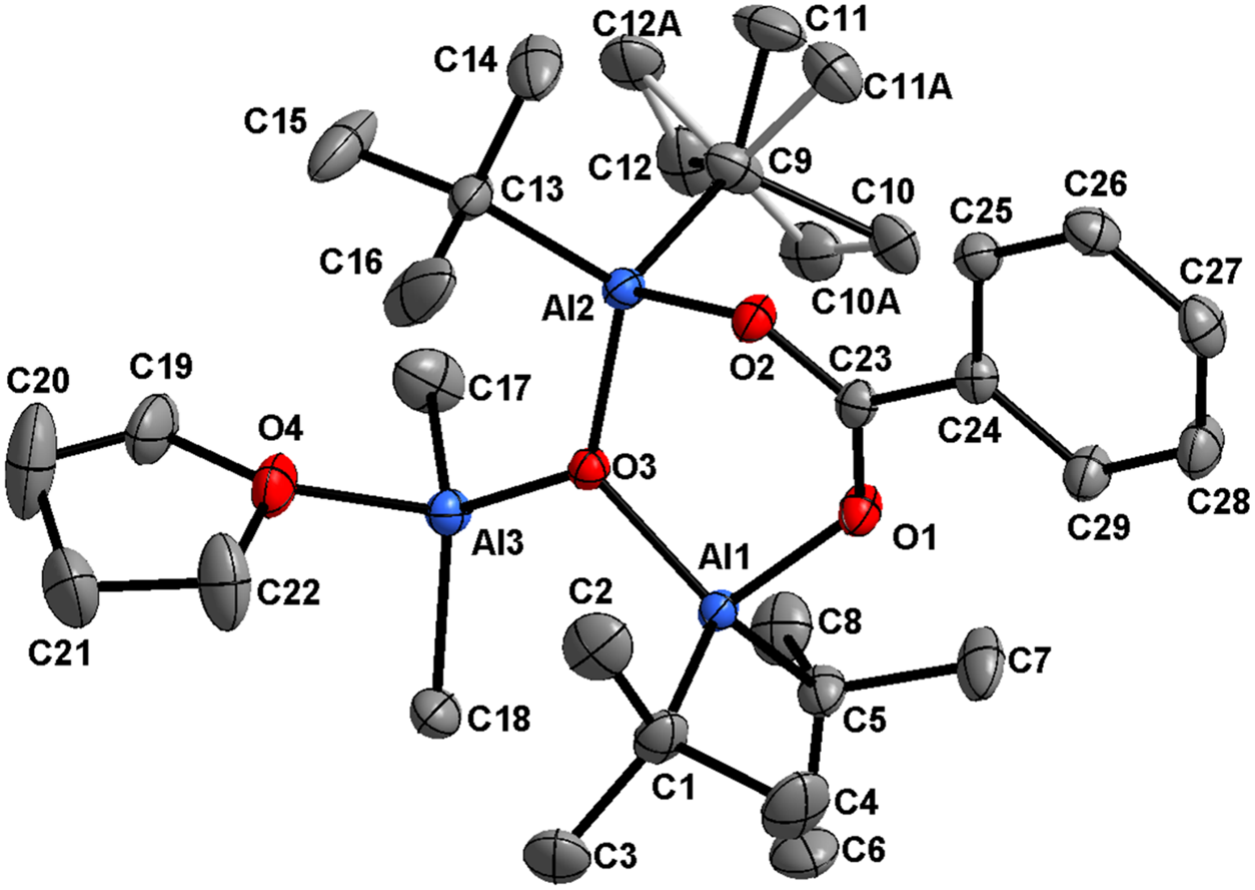
Thermal ellipsoid plot (50% probability) of compound **7**. Hydrogen atoms have been omitted for the sake of clarity. Positions of one *t*-Bu group atoms are disordered. Selected bonds (Å) and angles (°): C(9)–Al(2) 2.013(2), C(13)–Al(2) 2.007(2), C(17)–Al(3) 1.956(2), C(18)–Al(3) 1.957(2), C(23)–O(1) 1.265(2), C(23)–O(2) 1.269(2), C(23)–C(24) 1.484(2), Al(1)–O(3) 1.852(1), Al(1)–O(1) 1.863(1), Al(2)–O(3) 1.847(1), Al(2)–O(2) 1.856(1) Al(3)–O(3) 1.806(1), Al(3)–O(4) 1.914(1), O(3)–Al(1)–O(1) 101.15(5), O(3)–Al(2)–O(2) 102.71(5), O(3)–Al(3)–O(4) 107.58(6), C17 Al3 C18 116.53(9), C(23)-O(1)–Al(1) 131.8(1), C(23)–O(2)–Al(2) 127.3(1), Al(3)–O(3)–Al(2) 123.66(6), Al(3)–O(3)–Al(1) 116.03(6), Al(2)–O(3)–Al(1) 119.60(6), O(1)–C(23)–O(2) 122.4(1), O(1)–C(23)–C(24) 118.9(1), and O(2)–C(23)–C(24) 118.7(1).

**Figure 3:**
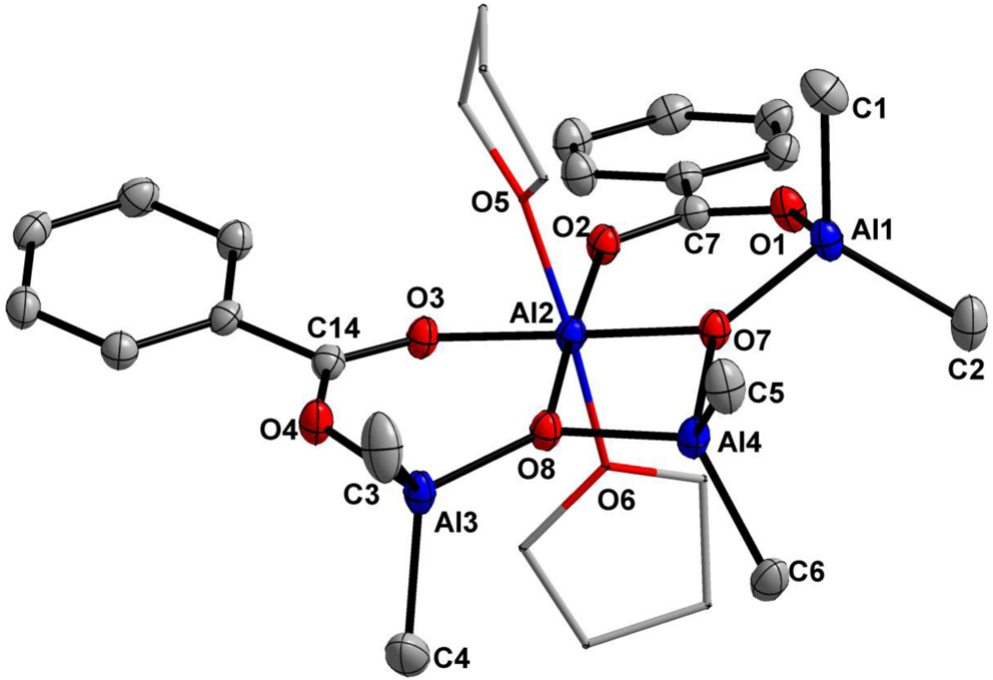
Thermal ellipsoid plot (50% probability) of compound **8**. Hydrogen atoms have been omitted for the sake of clarity. Selected bonds (Å) and angles (°): Al(1)–O(1) 1.861(1), Al(1)–O(7) 1.769(1), Al(2)–O(2) 1.901(1), Al(2)–O(3) 1.888(1), Al(2)–O(5) 1.970(1), Al(2)–O(6) 1.993(1), Al(2)–O(7) 1.830(1), Al(2)–O(8) 1.848(1), Al(3)–O(4) 1.859(1), Al(3)–O(8) 1.768(1), Al(4)–O(7) 1.813(1), Al(4)–O(8) 1.821(1), C(7)–O(1) 1.265(2), C(7)–O(2) 1.269(2), C(14)–O(3) 1.266(2), C(14)–O(4) 1.267(2), O(5)–Al(2)–O(6) 172.40(4), O(2)–Al(2)–O(5) 89.06(4), O(2)–Al(2)–O(6) 86.16(4), O(3)–Al(2)–O(2) 85.17(4), O(3)–Al(2)–O(5) 85.26(4), O(3)–Al(2)–O(6) 88.45(4), O(1)–C(7)–O(2) 124.5(1), O(3)–C(14)–O(4) 124.4(1).

All manipulations were carried out by using standard Schlenk techniques under an inert gas atmosphere. Methylene dichloride was deacidified with basic Al_2_O_3_ and distilled over P_2_O_5_ under argon. ^1^H and ^13^C NMR spectra were obtained on a Mercury-400BB Varian spectrometer. Chemical shifts were referenced to the residual proton signals of CDCl_3_ (7.26 ppm). ^13^C NMR spectra were acquired at 100.60 MHz (standard: chloroform ^13^CDCl_3_, 77.20 ppm). All NMR spectra can be found in the Supporting Information (Figures 1S–4S). Anhydrous AlCl_3_ was obtained from ABCR Company. It was sublimed under vacuum before reactions. *t*-BuMgCl (2.0 M solution in Et_2_O) and trimethylaluminum Me_3_Al were from Aldrich. *t*-Bu_3_Al etherate and *t*-Bu_3_Ga were synthesized according to the literature.^48,49^**Caution***! Trimethylaluminum is extremely pyrophoric. It must be handled using proper needle and syringe techniques under argon.*

### 2.2 Synthesis of Di-*t*-butylgallium Carboxylates (According to the Modified Procedure Described in (49))

To a solution of carboxylic acid (1 mmol, 1.22 g of benzoic acid, or 1.02 g of pivalic acid, or 2.12 g of diphenylacetic acid) in 15 cm^3^ of THF cooled to 0 °C, a solution of *t*-Bu_3_Ga (1 mmol, 2.41 g) in 15 cm^3^ of THF was added. After 2 h, the reaction mixture was warmed to room temperature. The solvent was removed under vacuum, and a white solid was obtained.

Di-*t*-butylgallium Benzoate: Mp.: 202–205 °C.

Di-*t*-butylgallium Pivalate: mp.: 83–86 °C, ^1^H NMR δ: 1.27 (18H, s, (CH_3_)_3_C), 1.01 (36H, s, GaC(CH_3_)_3_). ^13^C NMR δ: 41.64 ((CH_3_)_3_CCOO), 29.76 (GaC(CH_3_)_3_), 28.12 ((CH_3_)_3_CCOO), 23.95 (GaC(CH_3_)_3_) ppm.

Di-*t*-butylgallium Diphenylacetate: ^1^H NMR δ: 7.40 (8H, m, H_aromat_), 7.33 (4H, m, H_aromat_), 7.26 (8H, m, H_aromat_), 5.08 (2H, s, (C_6_H_5_)_2_CH), 0.79 (36H, s, GaC(CH_3_)_3_). ^13^C NMR δ: 181.56 (COO), 138.96, 129.10, 128.75, 128.13 (C_aromat_), 60.92 ((C_6_H_5_)_2_CH), 29.27 (GaC(CH_3_)_3_), 24.04 (GaC(CH_3_)_3_) ppm.

### 2.3 Synthesis of Carboxylatoaluminum- and Carboxylatogallium Hydroxides

#### 2.3.1 Method a

To a solution of 1 mmol of *t*-Bu_3_M (M = Al, Ga) in 15 cm^3^ of THF cooled to −78 °C (for aluminum compounds) or 0 °C (for gallium compounds), a solution of 0.5 mmol of carboxylic acid and 0.5 mmol of water (0.5 mmol, 0.009 g) in 15 cm^3^ of THF was slowly added dropwise with continuous stirring. After 24 h, the postreaction mixture was concentrated and placed at −20 °C (aluminum compounds) and 7 °C (gallium compounds). After a few days, a crystalline white solid was precipitated.

#### 2.3.2 Method b

To a solution of 1 mmol of *t*-Bu_2_MOOCR (where M = Al, Ga; R = C_6_H_5_, or *t*-Bu, or (C_6_H_5_)_2_(H)C) in 15 cm^3^ of THF cooled to 0 °C, a solution of 1 mmol of *t*-Bu_3_M in 5 cm^3^ of THF was added. Then, a solution of water (1 mmol, 0.018 g) in 5 cm^3^ of THF was added. The next day, the solvent from the reaction mixture was distilled off to give a white solid.

#### 2.3.3 Method c

To a solution of 1 mmol of *t*-Bu_3_M (0.272 g of *t*-Bu_3_Al·OEt_2_, or 0.241 g of *t*-Bu_3_Ga) in 15 cm^3^ of THF cooled to – 78 °C, a solution of 1 mmol of water (0.018 g) in 5 cm^3^ of THF was added. Within 2 h, the reaction mixture was warmed to room temperature, and the solvent was distilled off in *vacuo*. *t*-Bu_2_MOH was obtained as a white solid. Then, a solution of *t*-Bu_2_MOH (0.5 mmol, 0.079 g) in 5 cm^3^ of THF was placed at −78 °C and a solution of *t*-Bu_3_Al·OEt_2_ in THF (0.5 mmol, 0.136 g, 5 cm^3^ of THF) was added. In the last step, a solution of 0.5 mmol of carboxylic acid in 5 cm^3^ of THF was slowly added dropwise to the reaction mixture. The mixture was warmed to room temperature over 2 h, and the solvent was distilled off in vacuo. Crystalline solids of **1–6** were obtained from hexane/THF solutions at −20 °C.

#### 2.3.4 Methods d

To a solution of 1 mmol of *t*-Bu_2_MOH (M = Al, Ga) in 10 cm^3^ of THF, a solution of 1 mmol of *t*-Bu_2_MOOCR (where M = Al, Ga; R = C_6_H_5_, *t*-Bu, or (C_6_H_5_)_2_(H)C) in 15 cm^3^ of THF was added at room temperature. The next day, the solvent from the reaction mixture was distilled off to give white solids of **1–6**.

Yields of compounds **1–6** are provided in Table 1S.

##### 2.3.4.1 [PhCO_2_Al_2_(*t*-Bu)_4_OH]·THF (**1**)

^1^H NMR (Figure 1S) δ: 8.15 (2H, m, H_aromat_), 7.69 (1H, m, H_aromat_), 7.52 (2H, m, H_aromat_), 5.27 (1H, s, OH), 3.84 (m, THF), 1.9 (m, THF), 0.97 (36H, s, (CH_3_)_3_CAl). ^13^C NMR δ: 175.97 (COO), 135.04, 131.13, 130.96, 128.78 (C_aromat_), 68.37 (THF), 30.27, 30.20 ((CH_3_)_3_CAl), 25.46 (THF), 15.06 (broad, (CH_3_)_3_CAl) ppm.

IR (cm^–1^): 3373 (broad), 3067 (w), 2969 (m), 2871 (w), 1602 (s), 1558 (s), 1428 (s), 1363 (m), 1205 (m), 1051 (m), 1026 (m), 906 (m), 845 (m), 719 (s), 666 (s), 531 (s).

Mp.: 156 °C.

##### 2.3.4.2 [Ph_2_C(H)CO_2_Al_2_(*t*-Bu)_4_OH]•THF (**2**)

^1^H NMR (Figure 2S) δ: 7.43–7.30 (10H, m, H_aromat_), 5.55 (1H, s, OH), 5.15 (1H, s, Ph_2_CH), 3.83 (m, THF), 1.89 (m, THF), 0.85 (36H, s, (CH_3_)_3_CAl). ^13^C NMR δ: 183.79 (COO), 137.29, 128.92, 128.73, 128.51, 127.85, 127.63 (C_aromat_), 68.40 (THF), 59.70 (Ph_2_CH), 30.18, 30.11 ((CH_3_)_3_CAl), 25.42 (THF) (14.76 (CH_3_)_3_CAl) ppm.

IR (cm^–1^): 3370 (very broad), 2969 (w), 2360 (w), 2338 (w), 1595 (m), 1582 (m), 1493 (w), 1425 (m), 1364 (m), 1201 (m), 1031 (w), 907 (m), 848 (m), 743 (m), 696 (s), 646 (s), 475 (s).

Mp.: 111.5 °C.

##### 2.3.4.3 [*t*-BuCO_2_Al_2_(*t*-Bu)_4_OH]·THF (**3**)

^1^H NMR (Figure 3S) δ: 3.64 (m, THF), 3.50 (1H, s, OH) 1.90 (m, THF), 1.29 (9H, s, (CH_3_)_3_CCOO), 0.93 (36H, s, (CH_3_)_3_CAl). ^13^C NMR δ: 68.62 (THF), 30.38 ((CH_3_)_3_CAl), 27.50 (CH_3_)_3_CCOO, 25.60 (THF) ppm. Due to the poor solubility of compound **3**, no other carbon signals were observed.

IR (cm^–1^): 3662 (w), 3096 (w), 2974 (m), 2974 (m), 2944 (m), 2922 (m), 2867 (m), 2827 (s), 1553 (s), 1490 (s), 1464 (s), 1444 (s), 1364 (m), 1231 (m), 1191 (m), 1153 (m), 1046 (m), 1002 (w), 886 (m), 813 (s), 647 (s), 628 (s), 594 (s), 547 (s).

Mp.: 140.6 °C.

##### 2.3.4.4 [PhCO_2_Ga_2_(*t*-Bu)_4_OH]·THF (**4**)

^1^H NMR (Figure 4S) δ: 8.09 (2H, m, H_aromat_), 7.58 (1H, m, H_aromat_), 7.46 (2H, m, H_aromat_), 3.77 (m, THF), 1.87 (m, THF), 1.69 (1H, s, OH), 1.10 (36H, s, (CH_3_)_3_CGa). ^13^C NMR δ: 176.38 (COO), 133.30, 132.30, 130.39, 128.44 (C_aromat_), 68.13 (THF), 30.37 ((CH_3_)_3_CGa), 25.67 (THF), 24.10 ((CH_3_)_3_CGa) ppm.

IR (cm^–1^): 3629 (w), 3264 (m, broad), 2949 (m), 2926 (m), 2869 (m), 2836 (m), 2702 (w), 1596 (m), 1550 (s), 1494 (w), 1466 (m), 1437 (m), 1417 (s), 1359 (m), 1315 (w), 1176 (w), 1119 (w), 1070 (m), 1049 (m), 1026 (m), 1011(m), 938 (m), 891 (m), 842 (m), 815 (s), 715 (s), 679, (s), 599 (m), 530 (s), 428 (m).

Mp.: 113–115 °C.

##### 2.3.4.5 [Ph_2_C(H)CO_2_Ga_2_(*t*-Bu)_4_OH]·THF (**5**)

^1^H NMR (Figure 5S) δ: 7.44–7.25 (10H, m, H_aromat_), 5.06 (1H, s, Ph_2_CH), 3.76 (m, THF), 1.86 (m, THF), 1.44 (1H, s, OH), 0.97 (36H, s, (CH_3_)_3_CGa). ^13^C NMR δ: 182.70 (COO), 138.92, 128.83, 128.85, 127.10 (C_aromat_), 69.97 (THF), 60.73 (Ph_2_C), 30.05 ((CH_3_)_3_CGa), 25.54 (THF), 23.78 ((CH_3_)_3_CGa) ppm.

IR (cm^–1^): 3582 (w), 3261 (m, broad), 3089 (w), 3062 (w), 3029 (w), 2948 (m), 2927 (m), 2914 (m), 2868 (m), 2836 (s), 1568 (s), 1491 (m), 1466 (m), 1452 (m), 1406 (s), 1359 (m), 1253 (m), 1118 (m), 1049 (m), 917 (m), 815 (m), 739 (m), 729 (m), 702 (m), 693 (m), 646 (m), 543 (s).

Mp.: 72–75 °C.

##### 2.3.4.6 [*t*-BuCO_2_Ga_2_(*t*-Bu)_4_OH]·THF (**6**)

^1^H NMR (Figure 6S) δ: 3.76 (m, THF), 1.86 (m, THF), 1.27 (1H, s, OH), 1.23 (9H, s, (CH_3_)_3_CCOO), 1.05 (36H, s, ((CH_3_)_3_CGa)). ^13^C NMR δ: 189.67 (COO), 67.92 (THF), 41.04 ((CH_3_)_3_CCOO), 30.12 ((CH_3_)_3_CGa), 27.68 ((CH_3_)_3_CCOO), 25.49 (THF), 23.54 ((CH_3_)_3_CGa) ppm.

IR (cm^–1^): (all bands are broadened) 3337 (m, broad), 2959 (s), 2931 (s), 2874 (s), 2845 (s), 1597 (s), 1551 (s), 1484 (s), 1469 (s), 1459 (s), 1427 (s), 1398 (s), 1380 (s), 1361 (s), 1226 (s), 1197 (s), 1073 (w), 1055 (w), 1032 (w), 1013 (w), 939 (m), 919 (m), 894 (w), 817 (m), 785 (w), 688 (m), 662 (s), 611 (s), 441 (s).

Mp.: 68–72 °C

##### 2.3.4.7 [PhCO_2_Al_2_(*t*-Bu)_4_OAlMe_2_]·THF (**7**)

Compound **1** (0.420 g, 1 mmol) was dissolved in 15 cm of THF. To the solution was added 0.072 g (1 mmol) of Me_3_Al in 5 cm THF. After 2 days, the solvent was distilled from the postreaction mixture, and the product was examined by NMR (yield 505 g, 92%). After several days at 7 °C, a small amount of crystals suitable for X-ray studies precipitated from the solution in hexane/THF.

^1^H NMR (Figure 7S) δ: 8.18 (2H, dd, *J*_H–H_^3^ = 8.1 Hz, *J*_H–H_^4^ = 1.2 Hz, H_aromat_), 7.67 (1H, m, H_aromat_), 7.51 (2H, m, H_aromat_), 4.32 (m, THF, broad), 2.14 (m, THF, broad), 0.97 (36H, s, (CH_3_)_3_CAl), −0.47 (6H, s, AlCH_3_). ^13^C NMR δ: 176.22 (COO), 134.68, 131.22, 129.49, 128.73 (C_aromat_), 72.98 (THF), 31.72, 31.74 ((CH_3_)_3_CAl), 24.83 (THF), 16.95 ((CH_3_)_3_CAl), −6.98 (AlCH_3_) ppm.

IR (cm^–1^): 2950 (m), 2928 (m), 2868 (m), 2825 (s), 1598 (s), 1549 (s), 1498 (m), 1451 (s), 1439 (s), 1384 (m), 1311 (w), 1208 (m), 1199 (m), 1181 (m), 998 (m), 845 (m), 811 (m), 717 (s), 693 (s), 684 (s), 672 (s), 635 (s), 585 (s), 577 (s), 538 (s).

Mp.:128–131 °C.

##### 2.3.4.8 [(PhCOO)_2_Al_4_Me_6_O_2_]·2THF (**8**)

30 cm^3^ of Me_3_Al (0.216 g, 3 mmol) solution in THF was placed in the reactor and cooled to −78 °C. A solution of benzoic acid (0.122 g, 1 mmol) and water (0.018 g, 1 mmol) in 20 cm^3^ of THF was slowly added dropwise to the reactor. The mixture was then warmed to room temperature over 2 h. After distilling off the solvent and volatiles, product **8** was obtained almost quantitatively as a white solid (0.300 g, 96%).

^1^H NMR (Figure 8S) δ: 8.14 (4H, m, H_aromat_), 7.63 (2H, m, H_aromat_), 7.47 (4H, m, H_aromat_), 4.06 (m, THF, broad), 2.03 (m, THF, broad), −0.69 (6H, s, CH_3_Al), −0.80 (12H, s, CH_3_Al). ^13^C NMR δ: 174.73 (COO), 134.76, 134.19, 131,38, 130.74, 129.96,129.30, 128.62, 128.32 (C_aromat_), 70.87 (THF), 35.37 (THF), −8.83 (CH_3_Al, broad) ppm.

IR (cm^–1^): 2931 (w), 1597 (m), 1549 (m), 1497 (w), 1429 (s), 1186 (m), 1071 (w), 1023 (w), 866 (w), 772 (m), 722 (s), 671 (s), 528 (m).

Mp.: 102–105 °C.

#### 2.3.5 Reactions of Carboxylatogallium Hydroxides with Me_3_Al

The carboxylatogallium hydroxide (**4**, 0.578g, 1 mmol or **6**, 0.486 g, 1 mmol) was dissolved in 20 cm^3^ of THF. 10 cm^3^ of Me_3_Al (0.216 g, 3 mmol) solution in THF was added to the solution, and the reaction mixture was heated to 66 °C. After cooling to room temperature, the volatile substances were removed by distillation under vacuum. The ^1^H NMR spectrum of the reaction products of **4** with Me_3_Al is the same as the spectrum of compound **8** (Figure 9S). The reaction of compound **6** with Me_3_Al resulted in the formation of compound [(*t*-BuCOO)_2_Al_4_Me_6_O_2_]·2THF (**9**), as evidenced by the ^1^H NMR spectrum (Figure 10S): 4.15 (m, THF, broad), 2.10 (m, THF, broad), 1.20 (9H, s, (CH_3_)_3_C), −0.73 (6H, s, CH_3_Al), −0.90 (12H, s, CH_3_Al) ppm. Compound **9** was obtained as a white solid after distillation of the solvent and volatiles. The distillate was concentrated and examined by ^1^H NMR. The spectrum shows the signals of protons of *t*-BuGa at 0.97 ppm and protons of MeAl groups at −0.54 and −0.93 ppm (Figure 11S).

The attempt to crystallize compound **9** was unsuccessful.

#### 2.3.6 Probe the Polymerization of Olefins Using the Cp_2_ZrCl_2_/Compound 8 System as a Catalyst

A solution of **8** (0.262 g, 0.42 mmol in 5 cm^3^ of toluene, Al/Zr = 100) was added to a solution of Cp_2_ZrCl_2_ (0.005 g, 0.017 mmol in 10 cm^3^ of toluene). The resulting mixture was stirred for 30 min at room temperature. Olefin (neat, 85 mmol, 7.140 g of 1-hexene or 9.520 g of 1-octene, olefin/Zr = 5000) was added to the mixture and vigorously stirred at 60 ◦C for 20 h. After cooling to room temperature, 10% aq HCl was added to decompose the remaining compound **8**, and the organic layer was separated and poured into 100 cm^3^ of methanol. No polymer precipitate was observed.

### 2.4 X-ray Crystallography

The X-ray measurements of **1**, **3**, and **4** compounds were performed at 100(2) K on a Bruker D8 Venture Photon100 diffractometer equipped with a TRIUMPH monochromator and a Mo Kα fine focus sealed tube (λ = 0.71073 Å). Total frames were collected with a Bruker APEX2 program.^50^ The temperature of samples was 100(2) K. The frames were integrated with the Bruker SAINT software package^51^ using a narrow-frame algorithm. Data were corrected for absorption effects using the multiscan method (SADABS).^52^ The structures were solved and refined using the SHELXTL Software Package.^53^ The atomic scattering factors were taken from the International Tables.^54^ All hydrogen atoms were placed in calculated positions and refined within the riding model.

The crystals of **7** and **8** were selected under Paratone-N oil, mounted on the nylon loops, and positioned in the cold stream on the diffractometer. The X-ray data for the reported complexes were collected at 100(2)K on a SuperNova Agilent diffractometer and using Cu Kα radiation (λ = 1.54184 Å). The data were processed with CrysAlisPro.^55^ The crystal structures **7** and **8** were solved by direct methods using the SHELXT-97 program and were refined by full-matrix least-squares on F2 using the program SHELXL^56^ implemented in the Olex2 suite.^57^ All non-hydrogen atoms were refined with anisotropic displacement parameters. Hydrogen atoms were added to the structure model at geometrically idealized coordinates and refined as riding atoms.

Detailed crystallographic data are listed in Table 2S.

## 3 Results and Discussion

**Scheme 2:**
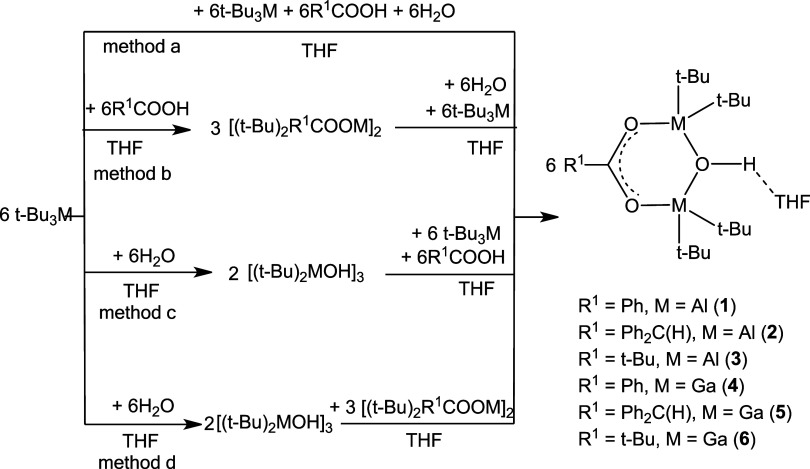
Synthesis of Carboxylatoaluminum- and Carboxylatogallium Hydroxides (**1–6**)

For many years, organoaluminum carboxylate compounds have been the subject of our intensive research.^58−60^ Recently, we have undertaken studies on the reactions of carboxylic acids with aluminum and gallium alkyl compounds and with water, which finally led us to carboxyalumoxanes. Reactions of *t*-Bu_3_M (M = Al, Ga) with selected monocarboxylic acids and water in a 2:1:1 molar ratio resulted in the formation of carboxylatoaluminum- and carboxylatogallium hydroxides [RCOOM_2_(*t*-Bu)_4_(OH)]·THF, where R = Ph, M = Al (**1**); R = Ph_2_C(H), M = Al (**2**); R = *t*-Bu, M = Al (**3**); R = Ph, M = Ga (**4**); R = Ph_2_C(H), M = Ga (**5**); R = *t*-Bu, M = Ga (**6**). The four synthetic approaches were employed (Scheme 2):

(a) A solution of one equivalent of carboxylic acid and one equivalent of H_2_O in THF was added to a solution of two equivalents of *t*-Bu_3_M in THF. Generally, water and carboxylic acid are very reactive toward aluminum- and gallium trialkyls; however, due to steric hindrance of the *t*-Bu groups, *t*-Bu_3_Al starts to react after heating the reaction mixture to about −20 °C, while the reaction of *t*-Bu_3_Ga takes place at about 0 °C. Very likely, carboxylic acid and water react simultaneously with *t*-Bu_3_M in a similar temperature range, leading to the dimeric metal carboxylate [RCOOM(*t*-Bu)_2_]_2_ and the adduct of di-t-butyl metal hydroxide with THF, [(*t*-Bu)_2_MOH]·THF, which finally provide compounds **1–6**.

(b) In the first stage, metal carboxylate [RCOOM(*t*-Bu)_2_]_2_ was obtained by the reaction of carboxylic acid with *t*-Bu_3_M in a molar ratio of reagents of 1:1. Then, one equivalent of *t*-Bu_3_M was added to a solution of metal carboxylate [RCOOM(*t*-Bu)_2_]_2_ in THF at 0 °C, and finally, one equivalent of water was added. Under the reaction conditions, water only reacts with *t*-Bu_3_M to form [(*t*-Bu)_2_MOH]·THF, whereas the reaction of water with the metal carboxylate requires a higher temperature.

(c) In the first stage, [(*t*-Bu)_2_MOH]·THF was obtained by the reaction of *t*-Bu_3_M with H_2_O in a molar ratio of reagents of 1:1. Then, one equivalent of *t*-Bu_3_M was added to the solution of [(*t*-Bu)_2_MOH]·THF in THF at −78 °C and finally one equivalent of carboxylic acid was added. Upon slow heating of the reaction mixture, the carboxylic acid, which bears a more acidic proton compared to [*t*-Bu_2_MOH]·THF, first started to react with *t*-Bu_3_M. Usually, *t*-Bu_2_MOH hydroxides react with *t*-Bu_3_M to form alumoxanes or galloxanes with an M-O-M bond, but despite warming the reaction mixture to room temperature, [*t*-Bu_2_MOH]·THF remained unreacted because all of the *t*-Bu_3_M had reacted previously with the carboxylic acid.

(d) Compounds [RCOOM(*t*-Bu)_2_]_2_ and [(*t*-Bu)_2_MOH]·THF were synthesized in two separate reactions, isolated, and then a solution of the compound [RCOOM(*t*-Bu)_2_]_2_ in THF was added to a solution of the compound [*t*-Bu_2_MOH]·THF in THF in a 1:1 molar ratio of the reagents. Compounds **1–6** were formed by dissociation of the [RCOOM(*t*-Bu)_2_]_2_ dimers into monomers in the presence of [(*t*-Bu)_2_MOH]·THF as a strong Lewis acid and the formation of a bond between the oxygen atom of the RCOOM(*t*-Bu)_2_ monomer and the metal atom of [(*t*-Bu)_2_MOH]·THF. The use of pure starting materials [RCOOM(*t*-Bu)_2_]_2_ and [(*t*-Bu)_2_MOH]·THF in the synthesis by method (d) allows obtaining pure compounds **1–6**, which means better quality control of the obtained products in comparison with the methods (a)–(c). On the other hand, the one-step method (a) is simple and allows for the rapid obtaining of larger quantities of products of good purity.

Generally, independent of the synthesis methods, compounds **1–6** were obtained as almost pure substances, which was demonstrated by NMR spectroscopy, indicating that there is only a single form in each case. The signals of the hydroxyl group protons are observed in the ^1^H NMR spectra in different ranges, i.e., at 5.27, 5.55, and 3.50 ppm for aluminum derivatives **1–3**, and at 1.69, 1.44, and 1.27 ppm for gallium derivatives **4–6**, respectively. The signals of the *t*-BuAl-protons appeared in the range of 1.10 to 0.85 ppm depending on the compound as singlets, which indicates their equivalence within the appropriate compound. The integration ratios of the *t*-BuM-protons signals and those of the carboxylate units show approximately 36 *t*-BuM protons per carboxylate unit, which confirms the composition of the compounds **1–6** matching the formula [RCOOM_2_(*t*-Bu)_4_(OH)]·THF.

As for the reaction pathway of compound **1–6** formation, compounds [RCOOM(*t*-Bu)_2_]_2_ and *t*-Bu_2_MOH are most likely formed first as products of the reactions of carboxylic acid and *t*-Bu_3_M, and *t*-Bu_3_M with water, respectively. In the next step, these compounds combine, yielding carboxylatometal hydroxides **1–6** (Scheme 2). This is evidenced by the fact that the order of addition of the reagents does not affect the formation of carboxylatometal hydroxides **1–6** (methods a–c). Moreover, compounds **1–6** are formed quantitatively upon mixing metal carboxylates RCOOM(*t*-Bu)_2_ and di-t-butyl metal hydroxides *t*-Bu_2_MOH (method d).

The compounds **1**, **3**, and **4** were isolated as crystals suitable for X-ray measurements. Molecular structures of compounds **1**, **3**, and **4** are shown in Figures 12S, 1S, and 13S, respectively. Details of data collection and structure analysis are summarized in Table 2S (Supporting Information). The central part of the molecules is a six-membered-ring M_2_CO_3_ in a half-chair conformation. Molecules consist of one carboxylate unit, two metal atoms bonded to two *t*-Bu groups each, and an OH group forming a bridging bond between the metal atoms. The bridging OH groups form O–H···O hydrogen bonds with the oxygen atoms of THF molecules. The bond lengths between the metal atoms and the oxygen atom of the OH group are almost the same: Al(1)–O(1) 1.840(4) and Al(2)–O(1)-1.838 Å in **1**; Al(1)–O(1) 1.825(3) and Al(2)–O(1) 1.830(3) Å in **3**; and Ga(1)–O(2) 1.929(2) and Ga(2)–O(2) 1.933(1) Å in **4**. On the other hand, the C–O bond lengths in the COO carboxyl groups differ slightly, indicating that a shorter bond between the oxygen and carbon atoms has partially retained the character of a C=O double bond. The largest difference in C–O bond lengths was observed for compound **3**, in which the bond lengths are C(1A)-O(3) 1.236(3) and C(1A)-O(2) 1.294(3) Å, respectively (Figure 1).

**Scheme 3:**
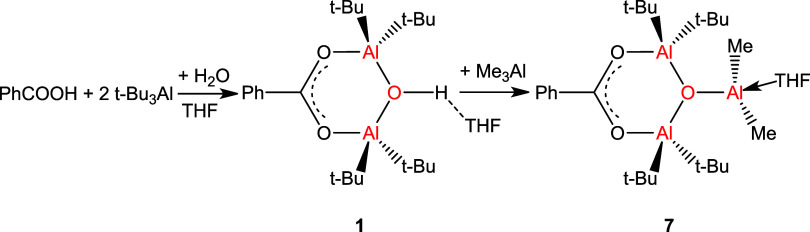
Two-Step Synthesis of the Carboxyalumoxane **7**

The presence of the OH group in compounds **1–6** prompted us to investigate their reactivity toward Me_2_Zn, Me_3_Ga, Me_3_In, *t*-Bu_3_Al, and *t*-Bu_3_Ga. At room temperature, no reactions occurred, but upon heating the reaction mixture up to the THF boiling point, a complicated mixture of products appeared in most cases. Only the reaction of carboxylatoaluminum hydroxides with Me_3_Al for a 1:1 molar ratio leads to the formation of carboxyalumoxanes. Reacting compound **1** with Me_3_Al, compound **7** was obtained, which contains a (*t*-Bu_2_Al)_2_OAlMe_2_ alumoxane motif supported by a carboxylate anion (Scheme 3). Compound **7** is “mixed” carboxyalumoxane with different alkyl groups bonded to aluminum atoms, which is driven by the two-step synthetic pathway, i.e., the necessity to generate a stable carboxylatoaluminum hydroxide only possible with sterically crowded alkyl substituents on the Al center. The second step, which involves the reaction of an acidic −OH proton with another R_3_Al unit, may take place in the case of smaller substituents (for example, R = Me) by their smoother approach to the carboxylatoaluminum hydroxide molecule that additionally interacts with the THF molecule by a hydrogen bond (see the description of the molecular structure of compounds **1**, **3**, and **4** above).

The ^1^H NMR spectrum of compound **7** reveals signals of aromatic protons (8.18–7.51 ppm), singlet protons of *t*-Bu groups at 0.97 ppm, singlet protons of Me groups at −0.47 ppm, and a lack of −OH proton signal. An integration ratio of 5.1:36,6:5.4 confirms the reaction of Me_3_Al with the −OH moiety, which corresponds to the structure of the product in Scheme 3. Compound **7** is the major product in the postreaction mixture after distillation of volatiles, as seen in the ^1^H NMR spectrum (Figure 7S).

Based on X-ray studies, it was found that molecule **7** contains a six-membered Al_2_CO_3_ ring, the same as in starting compound **1** (Figure 2). The μ-O fragment is now connected to the Me_2_Al moiety, and the tetrahedral coordination environment of the added Al center is complemented by a THF molecule. Thus, the molecular structure of compound **7** incorporates an alumoxane fragment supported by a carboxylate anion and could therefore be identified as a carboxyalumoxane. The benzoate moiety-support alumoxane motif in which Al(1) and Al(2) atoms are bonded to *t*-Bu groups, while Al(3) is bonded to two Me groups. The Al(1)–O(3) and Al(2)–O(3) bonds in the alumoxane moiety are of similar length (1.852(1) and 1.847(1) Å, respectively) and are slightly longer than the Al(3)–O(3) 1.806(1) Å bond.

**Scheme 4:**
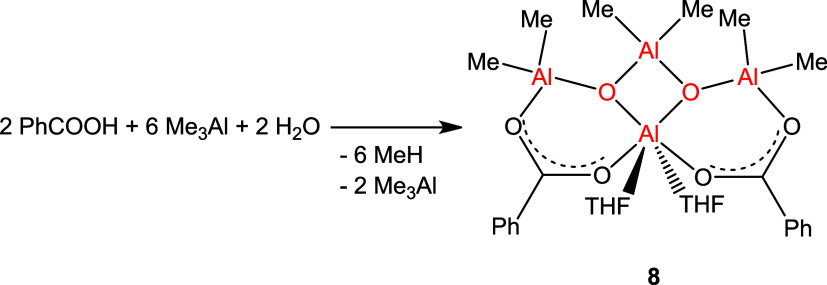
Synthesis of Carboxyalumoxane **8**

Attempting to obtain an analogous derivative containing only Me substituents on the aluminum center, keeping in mind the instability of carboxylatoaluminum hydroxide substituted with low-alkyl substituents, we further decided to use a one-step protocol reacting benzoic acid with Me_3_Al and water in the molar ratio 1:3:1 at −7* °C and slowly warming the reaction mixture to room temperature. After the solvent and volatiles were distilled off, solid carboxyalumoxane **8** was obtained almost quantitatively (Scheme 4).

The ^1^H NMR spectrum of **8** (Figure 8S) reveals multiplets of aromatic protons (8.14–7.47 ppm), two singlets at −0.69 and −0.80 ppm of MeAl protons in a 10.0:6.8:13.4 integration ratio, and two multiplets at 4.06 and 2.03 of THF protons, which suggests a structure of the product different from carboxyalumoxane **7** as confirmed by subsequent X-ray studies.

The molecular structure of carboxyalumoxane **8** was determined by single-crystal X-ray diffraction analysis and is demonstrated in Figure 3. The compound, different from carboxyalumoxane **7**, consists of an alumoxane unit Me_6_Al_4_O_2_ supported by two benzoate anions. Each of the tetrahedral Al(1), Al(3), and Al(4) aluminum atoms are bonded to two Me groups and two oxygen atoms, while the octahedral Al(2) aluminum center is surrounded by six oxygen atoms with four oxygen atoms O(2), O(3), O(7), O(8) derived by benzoate anions and two by THF molecules. The bonds of the Al(2) atom with the oxygen atoms of the carboxylate groups O(2) and O(3) [1.888(1) and 1.901(1) Å, respectively] are longer than the bonds of this atom with the μ_3_-oxygen atoms of O(7) and O(8) [1.530(1) and 1.848(1) Å, respectively], originating from the alumoxane unit. The lengths of the Al(2)–O(5) 1.970(1) Å and Al(2)–O(6) 1.993(1) Å bonds between the Al(2) atom and the oxygen atoms of the THF molecules indicate the coordinative nature of these bonds. The carboxylate’s C–O bond lengths, within the range of 1.265(2)–1.269(2) Å, are similar and indicate electron delocalization within the O–C–O fragments.

**Scheme 5:**
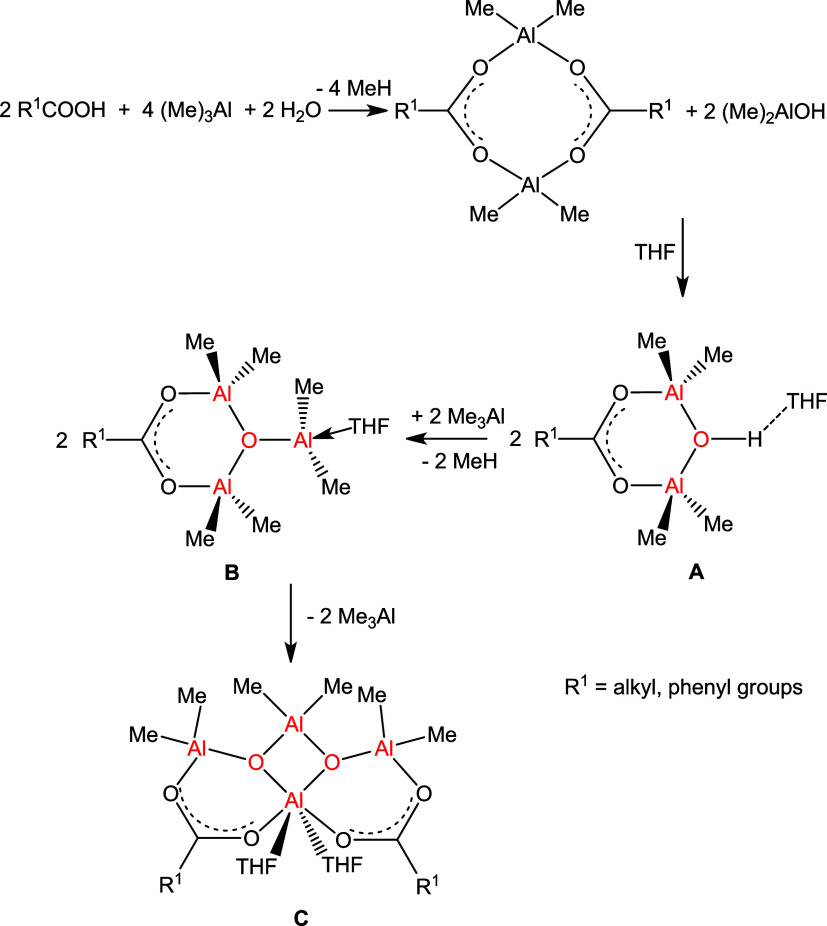
Proposed Pathway for Carboxy Methylalumoxanes Synthesis

In accordance with verified synthetic pathways to carboxylatoaluminum hydroxides (Scheme 2) and the isolation of *tert*-butyl-substituted carboxyalumoxane **7**, we proposed a sequence of reactions resulting in compound **8** (Scheme 5). In the first step, the reaction of Me_3_Al with carboxylic acid and water gives the well-known dimeric methylaluminum carboxylate [R^1^CO_2_AlMe_2_]_2_ and dimethylaluminum hydroxide. Being present together in the reaction mixture, they subsequently react, forming carboxylatoaluminum hydroxide **A** with a structure revealed in compounds **1–3**, which further reacts with Me_3_Al to result in carboxyalumoxane **B** with a structure similar to compound **7**. The final product **C** is the result of a complex Schlenk equilibrium that takes place in the presence of a coordinating solvent (THF), and its formation is accompanied by the release of two Me_3_Al molecules per product molecule. Mimicking the stoichiometry presented in the final product, it would seem that the molar ratio of R^1^COOH/Me_3_Al/H_2_O of the substrates should be 1:2:1. However, our experiments have indicated that this fails since a complex mixture of products is formed; thus, three equivalents of Me_3_Al was used instead leading to an almost quantitative formation of product **8** (for benzoic acid). This proves that the carboxylate anions support the metallic center so that the final -product **C** can be obtained via the dimethylaluminum intermediate **B** in a controlled manner.

The reaction of Me_3_Al with benzoic acid and water, as shown in Scheme 5, differs significantly from that under nonhydrolytic conditions reported by Cramail et al.^46^ In the first stage of the nonhydrolytic reaction, Me_3_Al reacts with benzoic acid at a 1:1 stoichiometry in toluene at 20 °C, forming dimethylaluminum benzoate (PhCOOAlMe_2_). Further additions of Me_3_Al lead to the formation of the alumoxane species and aluminum alkoxide PhCMe_2_OAlMe_2_ as a result of the methylation of the carbon atom of the carboxyl group. A similar methylation process was not observed in the reaction of Me_3_Al with benzoic acid and water. The final product **8** is a carboxyalumoxane containing two benzoic acid residues in the molecule and an alumoxane skeleton trapped in them, while in the nonhydrolytic reaction, two compounds are formed, aluminum alkoxide and alumoxane. The difference in the course of both reactions results not only from the presence of water and Me_2_AlOH formation but also from different reaction conditions. The reaction of Me_3_Al with benzoic acid and water takes place under mild conditions at −78 °C, as evidenced by the intensive gas evolution, while the nonhydrolytic reaction is carried out at high temperatures of 20 °C and above, which allows the cleavage of Al–C bonds. The coordinating properties of THF significantly impact the reaction course and product A, B, and C stability.

**Scheme 6:**
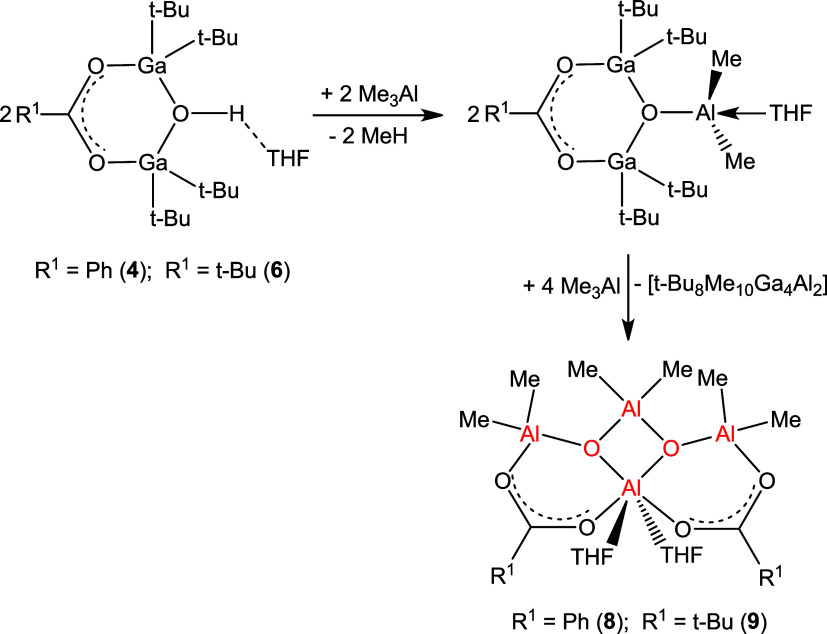
Reactions of Carboxylatogallium Hydroxides with Me_3_Al

In contrast to the reaction of carboxylatoaluminum hydroxide **1** with Me_3_Al yielding carboxyalumoxane **7**, the reactions of carboxylatogallium hydroxides **4–6** with Me_3_Al with a 1:1 molar ratio lead to a complicated mixture of products. Only the use of carboxylatogallium hydroxides and Me_3_Al in a 1:3 molar ratio provided well-defined products (Scheme 6). The ^1^H NMR spectrum of the reaction mixture of **4** with Me_3_Al (1:3) after the distillation of the volatile components unambiguously matches the spectrum measured for compound **8** and showed signals of compound **8** protons (Figure 9S). This result demonstrates that, in this approach, the formation of carboxyalumoxane **8** is the result of the reaction of the OH group with Me_3_Al and the exchange of (*t*-Bu)_2_Ga groups for Me_2_Al groups, concomitantly the carboxylate anions show a higher affinity toward the aluminum center versus the gallium center, hence a mixture of coproducts [*t*-Bu_8_Me_10_Ga_4_Al_2_] remains in the reaction mixture.

Similarly, the reaction between pivalatogallium hydroxide **6** and Me_3_Al in a 1:3 ratio yielded carboxyalumoxane product **9** (Scheme 6). The ^1^H NMR spectrum of the reaction mixture (Figure 10S) demonstrates two multiplets of THF protons (at 4.15 and 2.10 ppm), a singlet at 1.20 ppm of (CH_3_)_3_C protons, and two singlets at −0.73 and −0.90 ppm of Me group protons attached to aluminum atoms. The integration of the signals at 1.20:–0.73:–0.90 ppm (8.4:6.0:11.3) confirms the structure of carboxyalumoxane **9**. The spectrum does not show any signals of the protons of the *t*-BuGa groups at 1.05 ppm present in starting compound **6**, but signals appear at −0.73 and −0.90 ppm in the range typical for the signals of the MeAl group protons.

To investigate the activity of carboxyalumoxanes in the polymerization of alkenes, we performed test reactions using carboxyalumoxane **8** as a precatalyst, Cp_2_ZrCl_2_ as a cocatalyst, and 1-hexene and 1-octene as olefin substrates. The reactions were carried out under standard conditions: at 60 °C in toluene for 20 h, Zr/olefin = 1:5000, and Zr/Al = 1:100. The experimental results showed the lack of activity of compound **8** in the olefin polymerization process.

## 4 Conclusions

Although alumoxanes have been intensively studied for several decades due to their wide application in catalysis, materials engineering, and industry, their structure remains poorly understood. This is especially true for carboxyalumoxanes, which have been structurally characterized only in the case of derivatives with the bulk substituents at aluminum, such as *t*-Bu and (Me_3_Si)_3_C groups. In this work, we report the studies on the synthesis and structural characterization of carboxy methylalumoxanes incorporating a neutral Me_6_Al_4_O_2_ scaffold supported by two carboxylate anions. Furthermore, mapping out the pathway for the formation of these products, we developed a two-step method for the synthesis of well-defined carboxyalumoxanes containing a (*t*-Bu_2_Al)_2_OAlMe_2_ unit with two types of alkyl groups attached to aluminum atoms, which could further serve as a substrate for carboxyalumoxanes. We found that in the reaction of carboxylatogallium hydroxides with Me_3_Al, the *t*-Bu_2_Ga groups are exchanged for Me_2_Al units, which leads to the formation of carboxy methylalumoxanes. In the presented studies, a hydrolytic approach to the synthesis of carboxyalumoxanes was used, and their results can significantly help in understanding the individual stages of their formation.

## Supporting Information Available

^1^H NMR spectra of compounds; thermal ellipsoid plots of compounds **1** and **4;** and crystal data and data collection parameters for all compounds (PDF)
