# Supplementary material for: Stepwise Synthesis of Carboxyalumoxanes through Well-Defined Organoaluminum and Organogallium Carboxylatohydroxides Obtained by Controlled Hydrolysis
Source: Inorg Chem. 2025 May 1;64(18):8981–91. doi: 10.1021/acs.inorgchem.5c00367 (PMC12076546; doi:10.1021/acs.inorgchem.5c00367)
Supplement: Supplementary file 1 — ic5c00367_si_001.pdf [file ic5c00367_si_001.pdf]

Supporting Information

Stepwise Synthesis of Carboxyalumoxanes through Well-Defined Organoaluminum and Organogallium Carboxylatohydroxides Obtained by Controlled Hydrolysis

Wanda Ziemkowska[a*], Vadim Szejko[a], Bernadeta Prus[b], Paweł Socha[b], Michał K. Cyrański[b], Agnieszka Jastrzębska[c], Iwona Justyniak[d]

[a] Warsaw University of Technology, Faculty of Chemistry, Noakowskiego 3, 00-664 Warsaw, Poland,

[b] University of Warsaw, Department of Chemistry, Pasteura 1, 02-093 Warsaw, Poland,

[c] Warsaw University of Technology, Faculty of Mechatronics, św. Andrzeja Boboli 8, 02-526 Warsaw, Poland,

[d] Institute of Physical Chemistry Polish Academy of Sciences, Kasprzaka 44/52, 01-224 Warsaw, Poland

Corresponding author: E-mail for W. Ziemkowska: wanda.ziemkowska@pw.edu.pl

Content:

1. Fig. 1S. ^{1}H NMR spectrum of the compound **1**.
2. Fig. 2S. ^{1}H NMR spectrum of the compound **2**.
3. Fig. 3S. ^{1}H NMR spectrum of the compound **3**.
4. Fig. 4S. ^{1}H NMR spectrum of the compound **4**.
5. Fig. 5S. ^{1}H NMR spectrum of the compound **5**.
6. Fig. 6S. ^{1}H NMR spectrum of the compound **6**.
7. Fig. 7S. ^{1}H NMR spectrum of the compound **7**.
8. Fig. 8S. ^{1}H NMR spectrum of the compound **8**.
9. Fig. 9S. ^{1}H NMR spectrum of the post-reaction mixture of the compound $[PhCO_2Ga_2(t\text{-}Bu)_4OH]$•THF (**4**) with Me_3Al (1:3).
10. Fig. 10S. ^{1}H NMR spectrum of the post-reaction mixture of the compound $[t\text{-}BuCO_2Ga_2(t\text{-}Bu)_4OH]$•THF (**6**) with Me_3Al (1:3).
11. Fig. 11S. ^{1}H NMR spectrum of the distillate obtained from the reaction mixture of the compound **6** with Me_3Al.
12. Fig. 12S. Thermal ellipsoid plot (50% probability) of the compound **1**.
13. Fig. 13S. Thermal ellipsoid plot (50% probability) of the compound **4**.
14. Table 1S. Yields of compounds **1-6**.
15. Table 2S. Crystal data and data collection parameters for the compounds **1**, **2**, **4**, **7** and **8**.

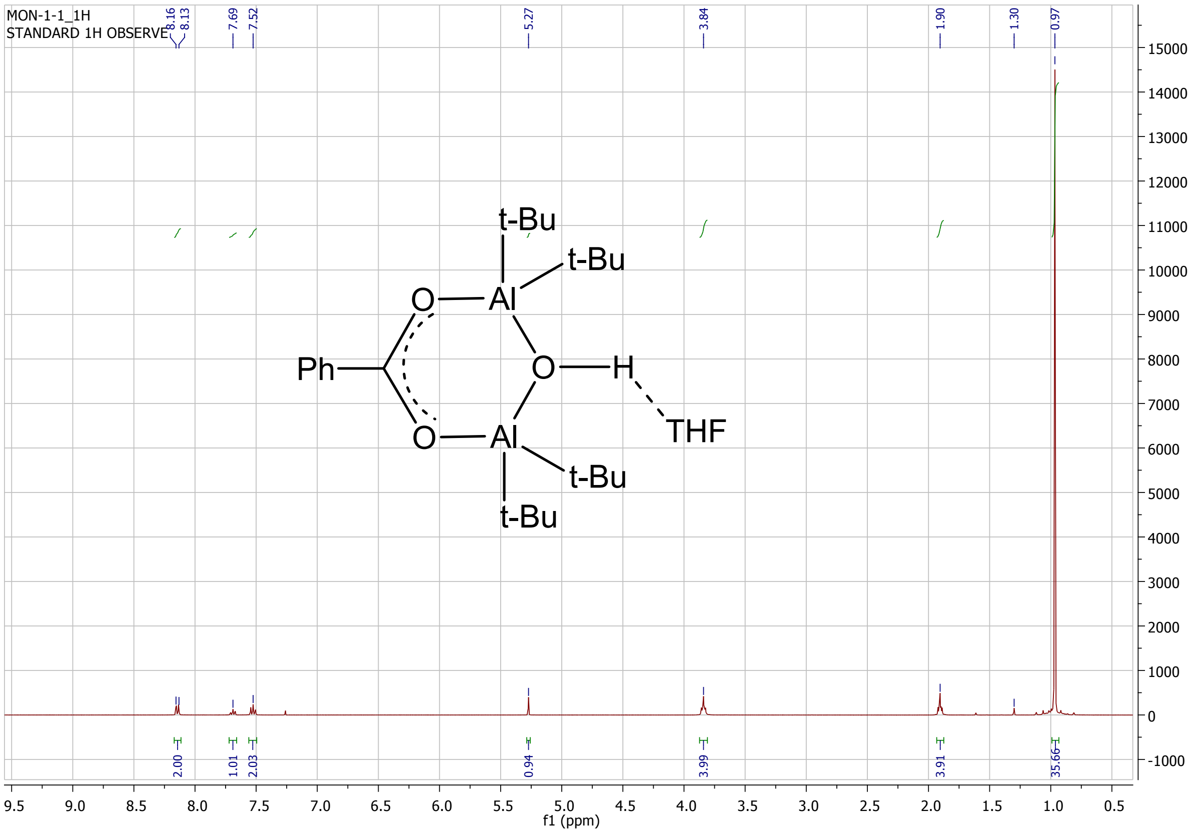

Fig. 1S. ^{1}H NMR spectrum of the compound $[PhCO_2Al_2(t\text{-}Bu)_4OH]$•THF (**1**): δ: 8.15 (m, H_{aromat}), 7.69 (m, H_{aromat}), 7.52 (m, H_{aromat}), 5.27 (s, OH), 3.84 (m, THF), 1.90 (m, THF), 0.97 (s, $(CH_3)_3CAl$) ppm.

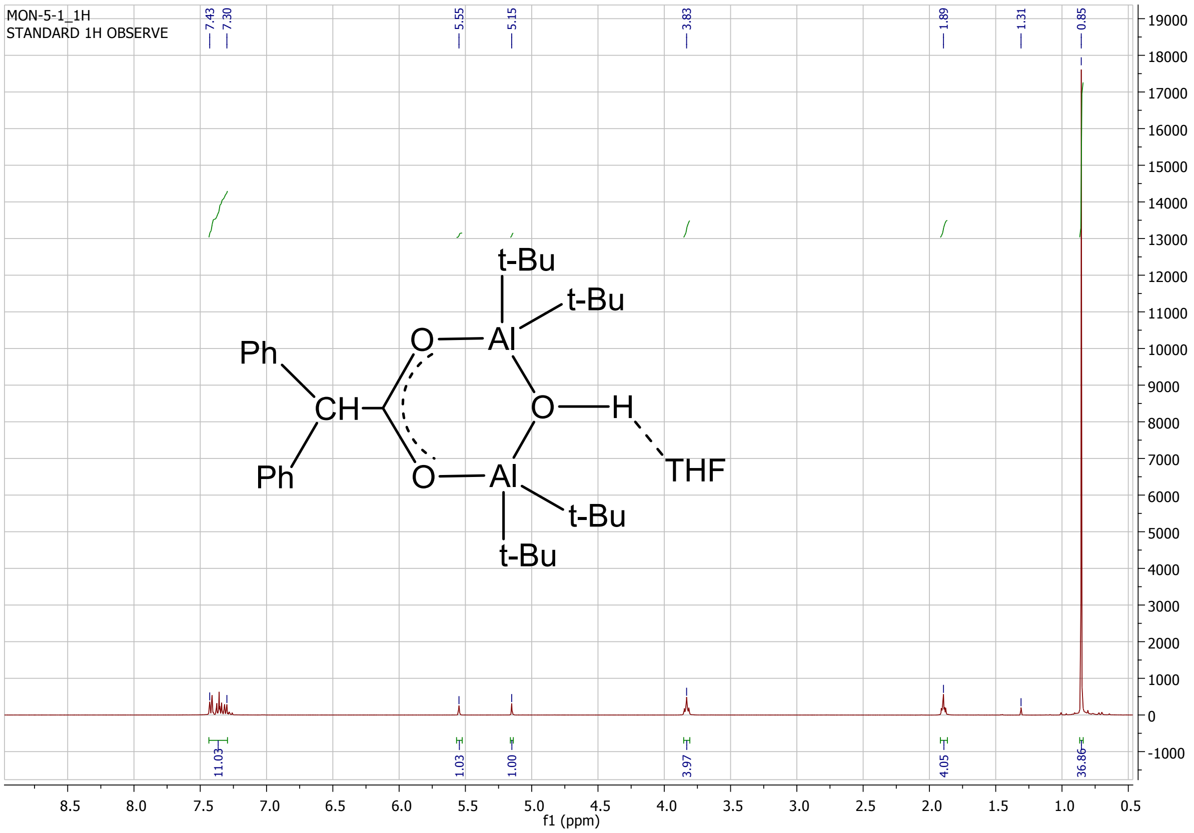

Fig. 2S. ^{1}H NMR spectrum of the compound $[Ph_2C(H)CO_2Al_2(t\text{-}Bu)_4OH]$•THF (**2**). δ: 7.43-7.30 (m, H_{aromat}), 5.55 (s, OH), 5.15 (s, Ph_2CH), 3.83 (m, THF), 1.89 (m, THF), 0.85 (s, $(CH_3)_3CAl$) ppm.

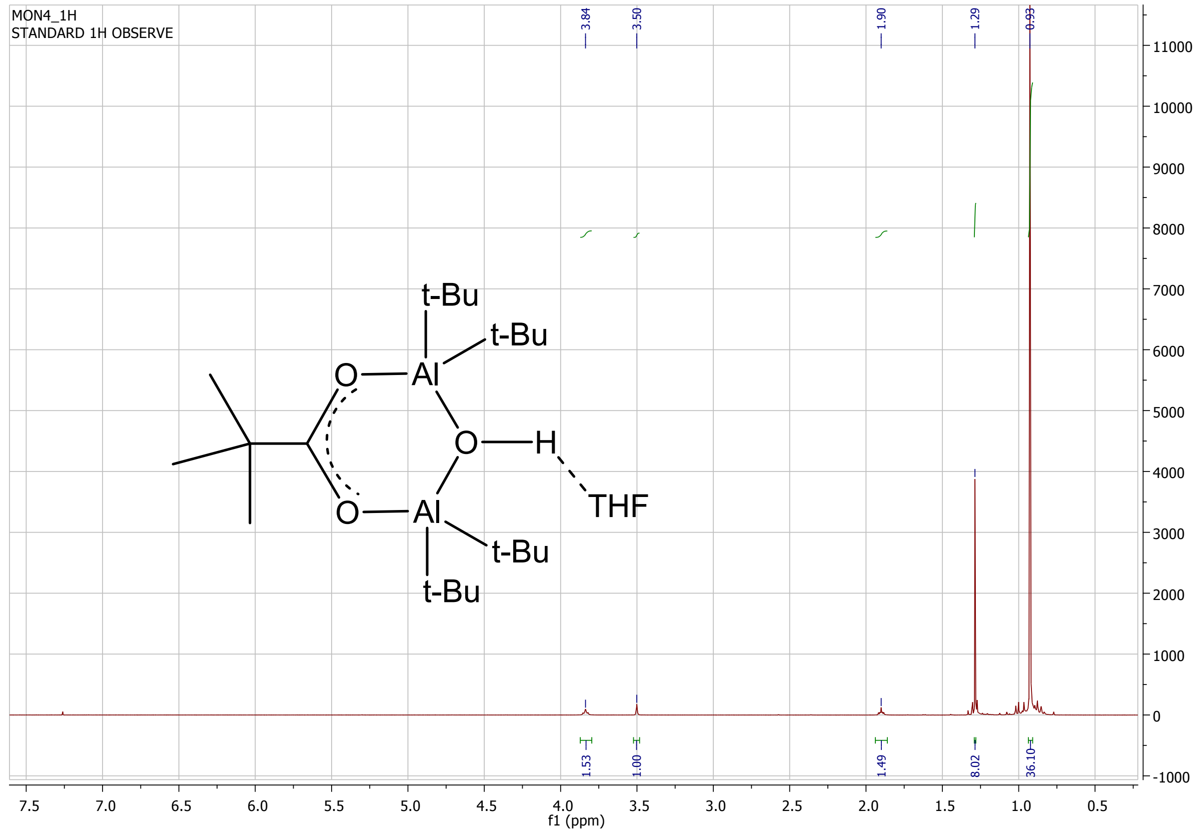

Fig. 3S. ^{1}H NMR spectrum of the compound [t-BuCO$_2$Al$_2$(t-Bu)$_4$OH]•THF (**3**). δ: 3.64 (m, THF), 3.50 (s, OH) 1.90 (m, THF), 1.29 (s, (CH$_3$)$_3$CCOO)), 0.93 (s, (CH$_3$)$_3$CAl) ppm.

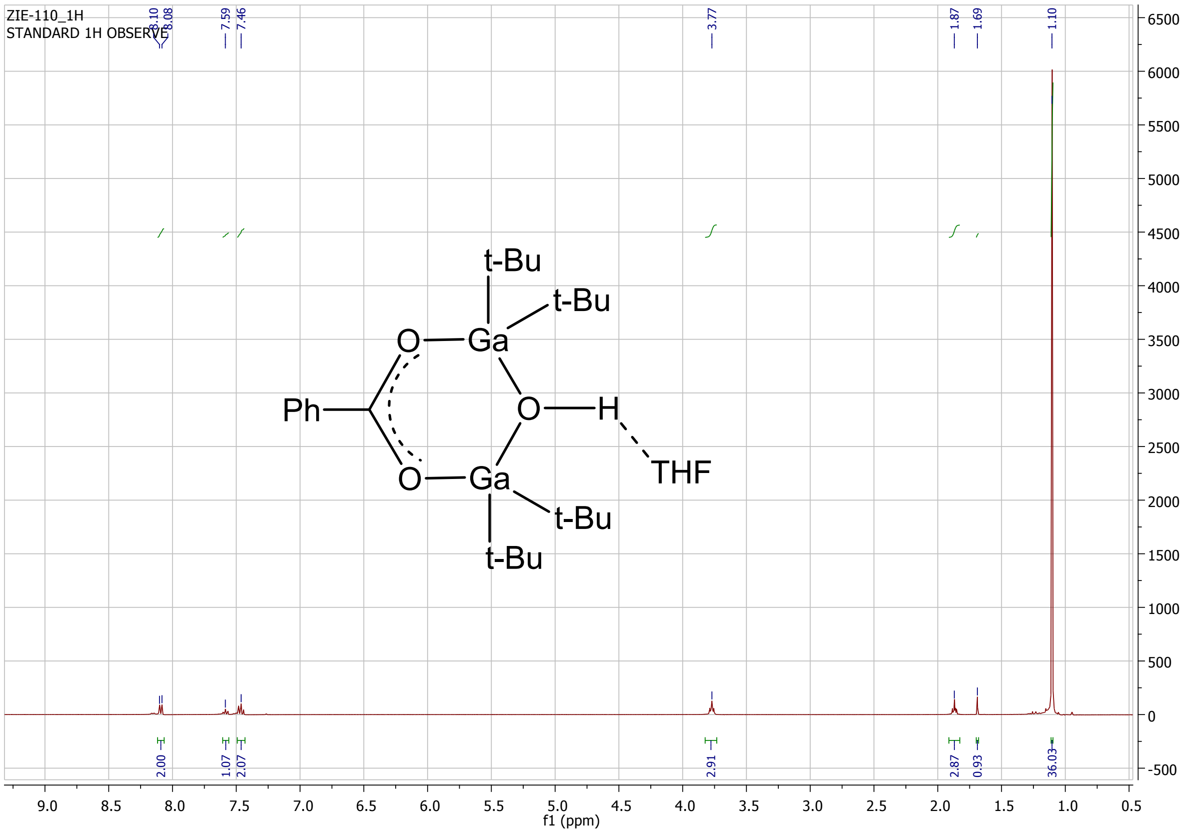

Fig. 4S. ^{1}H NMR spectrum of the compound $[PhCO_2Ga_2(t\text{-}Bu)_4OH]$•THF (**4**). δ: 8.09 (m, H_{aromat}), 7.58 (m, H_{aromat}), 7.46 (m, H_{aromat}), 3.77 (m, THF), 1.87 (m, THF), 1.69 (s, OH), 1.10 (s, $(CH_3)_3CGa$) ppm.

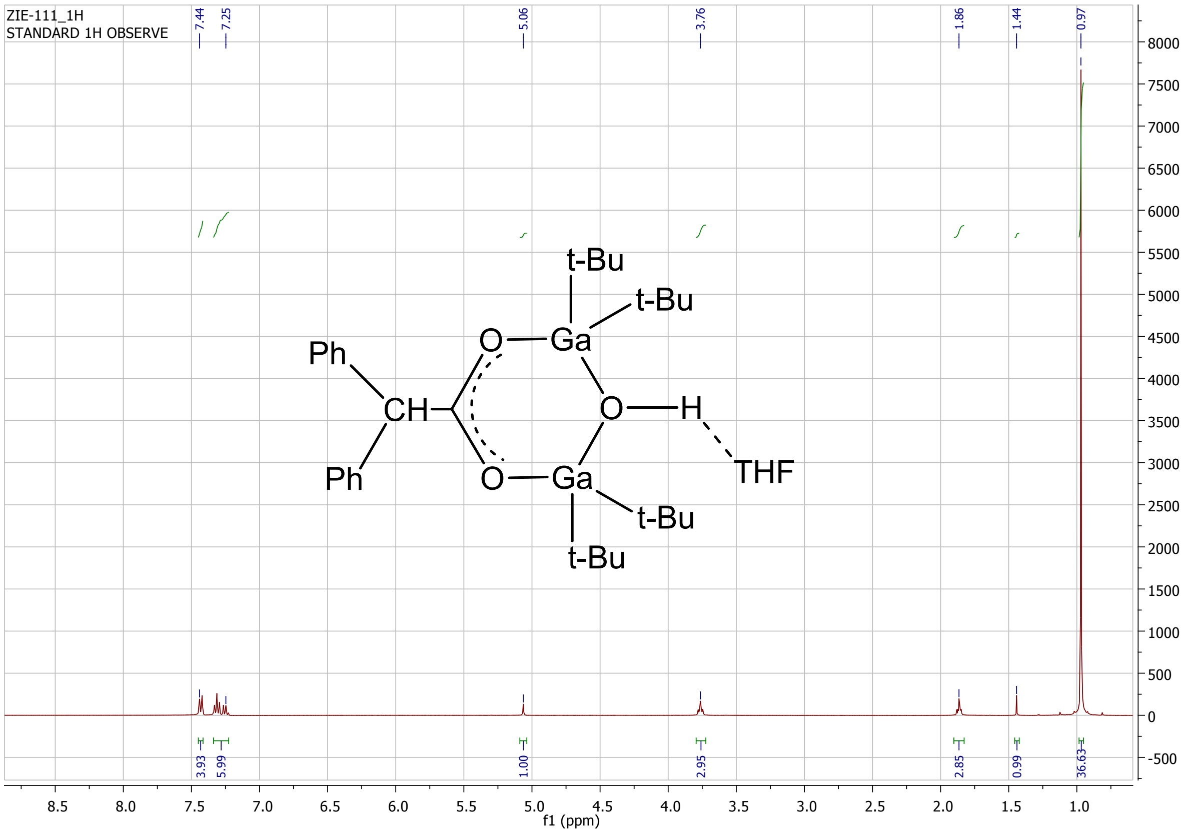

Fig. 5S. ^{1}H NMR spectrum of the compound [$Ph_2C(H)CO_2Ga_2(t\text{-}Bu)_4OH$]•THF (**5**). δ: 7.44-7.25 (m, H_{aromat}), 5.06 (s, Ph_2CH), 3.76 (m, THF), 1.86 (m, THF), 1.44 (s, OH), 0.97 (s, $(CH_3)_3CGa$) ppm.

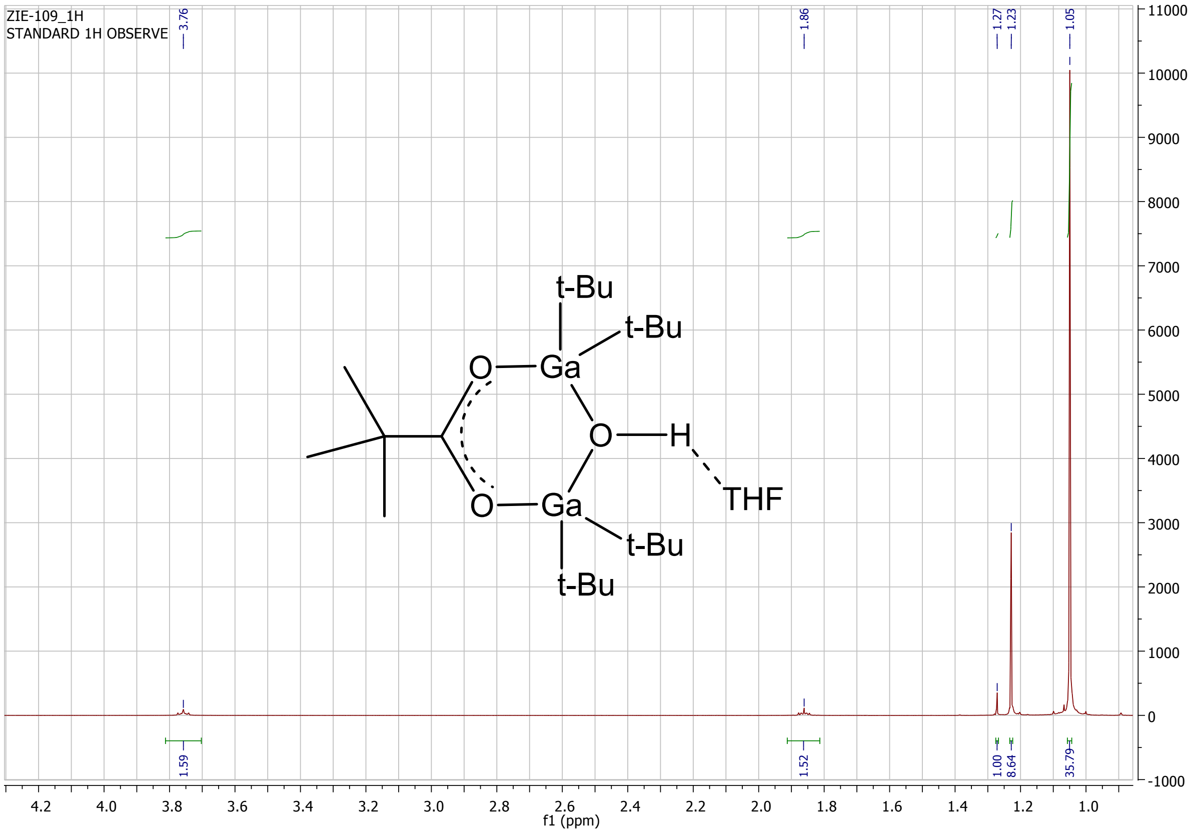

Fig. 6S. ^{1}H NMR spectrum of the compound [t-BuCO$_2$Ga$_2$(t-Bu)$_4$OH]•THF (**6**). δ: 3.76 (m, THF), 1.86 (m, THF), 1.27 (s, OH), 1.23 (s, (CH$_3$)$_3$CCOO), 1.05 (s, ((CH$_3$)$_3$CGa) ppm.

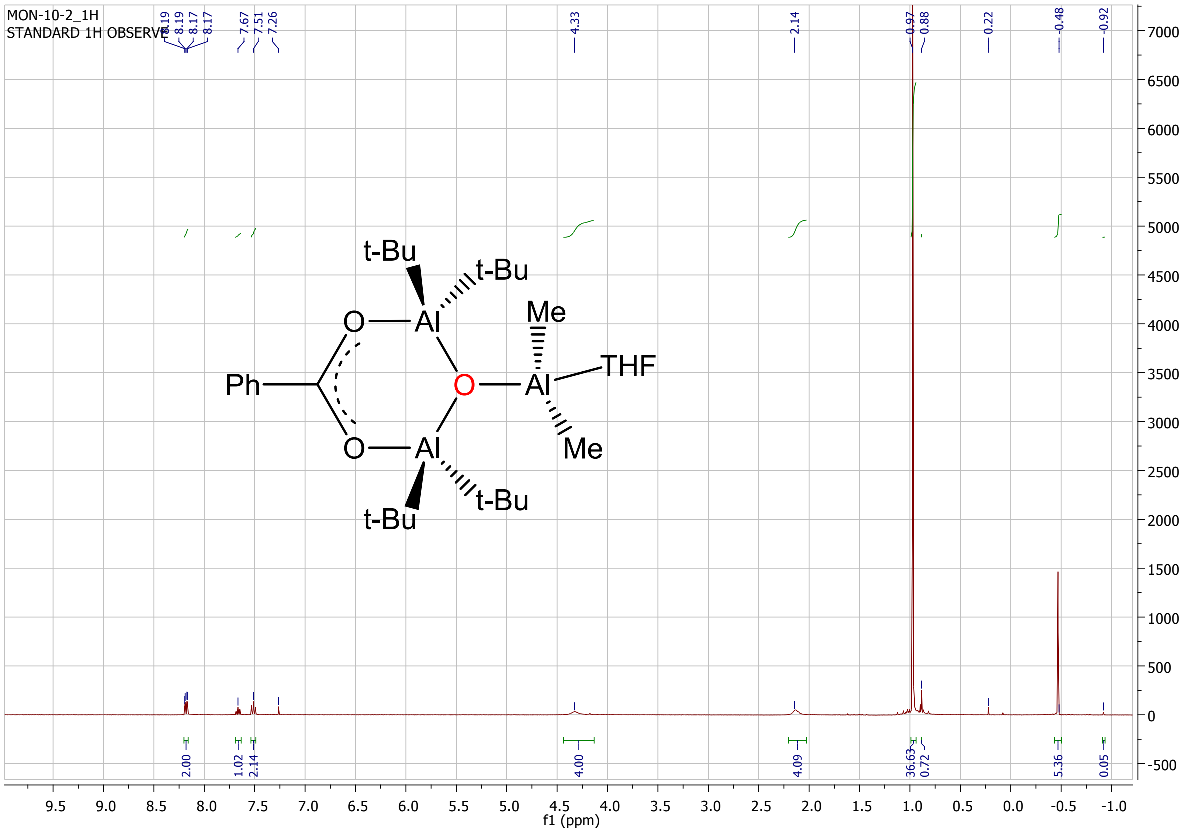

Fig. 7S. ^{1}H NMR spectrum of the compound $[PhCO_2Al_2(t\text{-}Bu)_4OAlMe_2]$•THF **7** (post-reaction mixture). 8.18 (H_{aromat}), 7.67 (m, H_{aromat}), 7.51 (m, H_{aromat}), 4.33 (m, THF, broad), 2.14 (m, THF, broad), 0.97 (s, $(CH_3)_3CAl$), -0.48 (s, $AlCH_3$). The signals at 0.88 ppm (tBuAl), at 0.22 ppm (silicon fat) and at -0.92 ppm (MeAl) were attributed to contaminations.

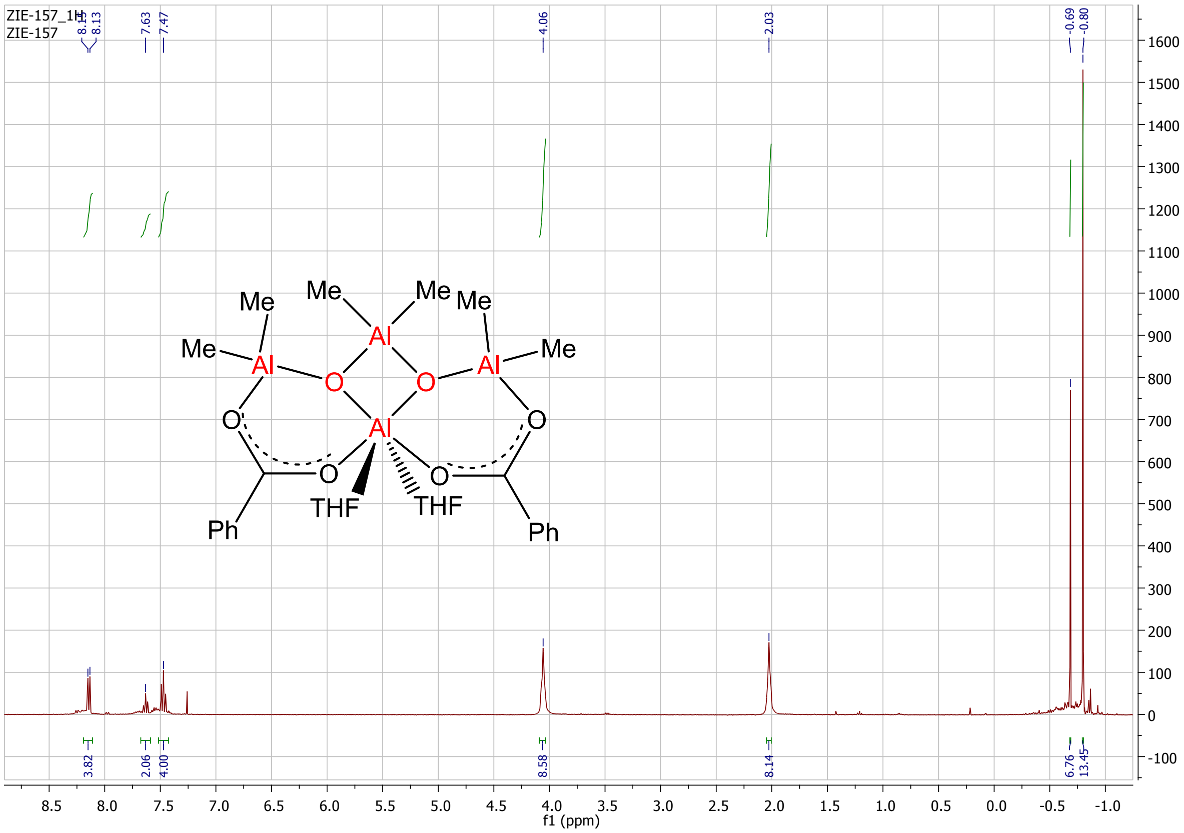

Fig. 8S. ^{1}H NMR spectrum of the compound $[(PhCOO)_2Al_4Me_6O_2]$•2THF (**8**) (post-reaction mixture). δ: 8.14 (m, H_{aromat}), 7.63 (m, H_{aromat}), 7.47 (m, H_{aromat}), 4.06 (m, THF, broad), 2.03 (m, THF, broad), -0.69 (s, CH_3Al), -0.80 (s, CH_3Al) ppm.

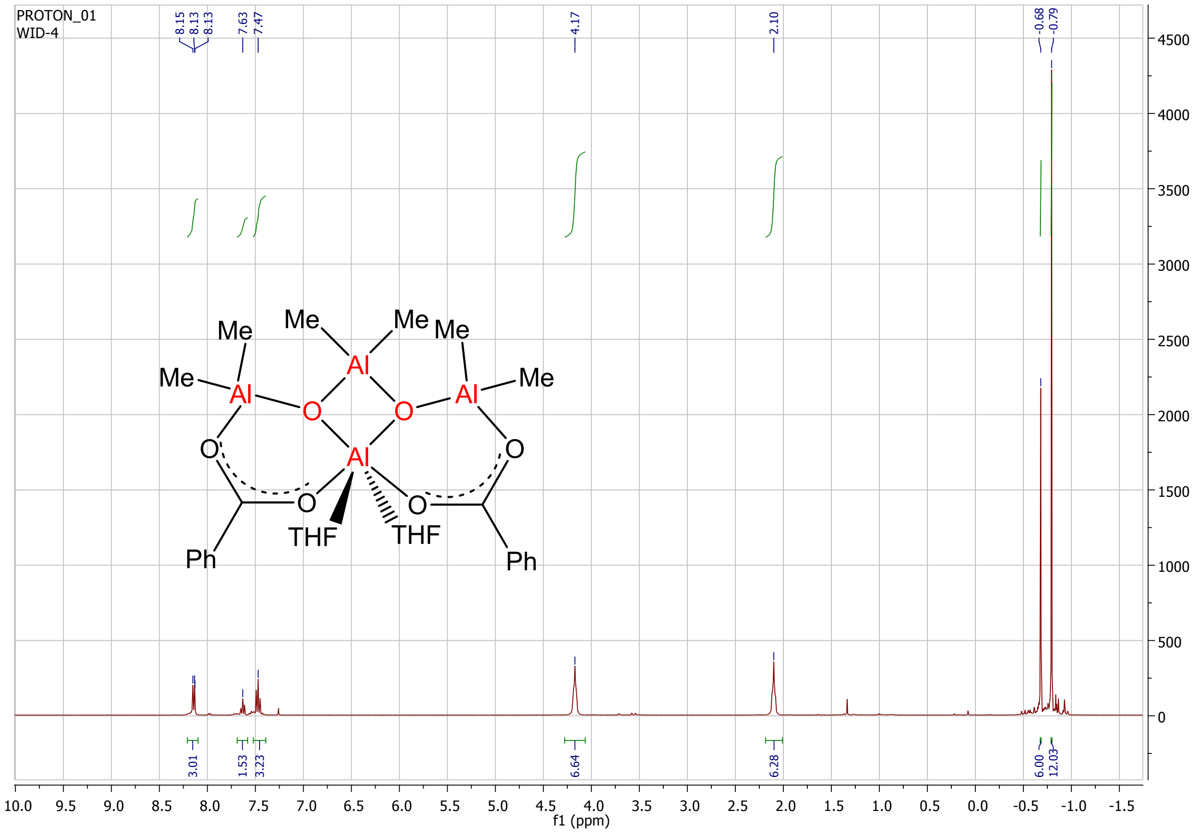

Fig. 9S. ^{1}H NMR spectrum of the post-reaction mixture of the compound $[PhCO_2Ga_2(t\text{-}Bu)_4OH]\bullet THF$ (**4**) with Me_3Al (1:3). The spectrum reveals signals of $[(PhCOO)_2Al_4Me_6O_2]\bullet 2THF$ (**8**). δ: 8.14 (m, H_{aromat}), 7.63 (m, H_{aromat}), 7.47 (m, H_{aromat}), 4.17 (m, THF, broad), 2.10 (m, THF, broad), -0.68 (s, CH_3Al), -0.79 (s, CH_3Al) ppm.

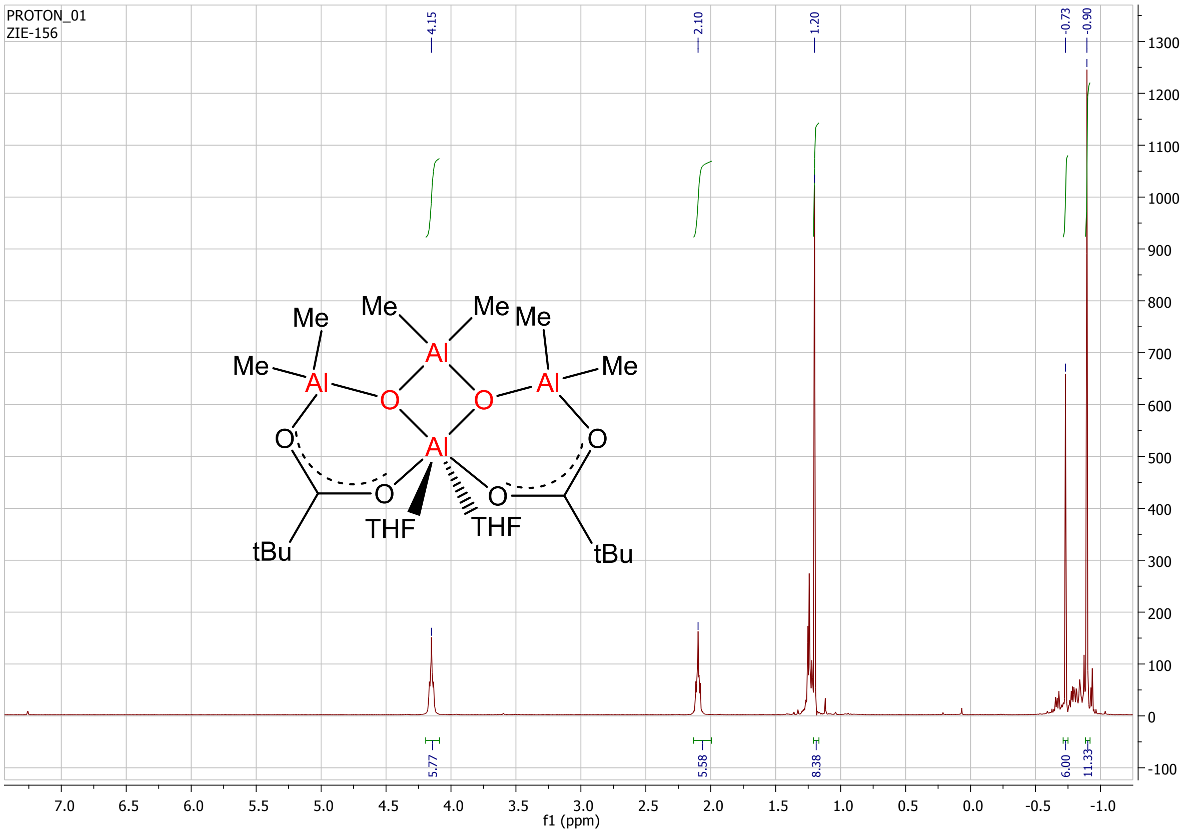

Fig. 10S. ^{1}H NMR spectrum of the post-reaction mixture of the compound [t-BuCO$_2$Ga$_2$(t-Bu)$_4$OH]•THF (**6**) with Me$_3$Al (1:3). The spectrum reveals signals of [(t-BuCOO)$_2$Al$_4$Me$_6$O$_2$]•2THF (**9**). δ: 4.15 (m, THF, broad), 2.10 (m, THF, broad), 1.20 (s, (CH$_3$)$_3$C), -0.73 (s, CH$_3$Al), -0.90 (s, CH$_3$Al) ppm. Other signals are the signals of contaminations.

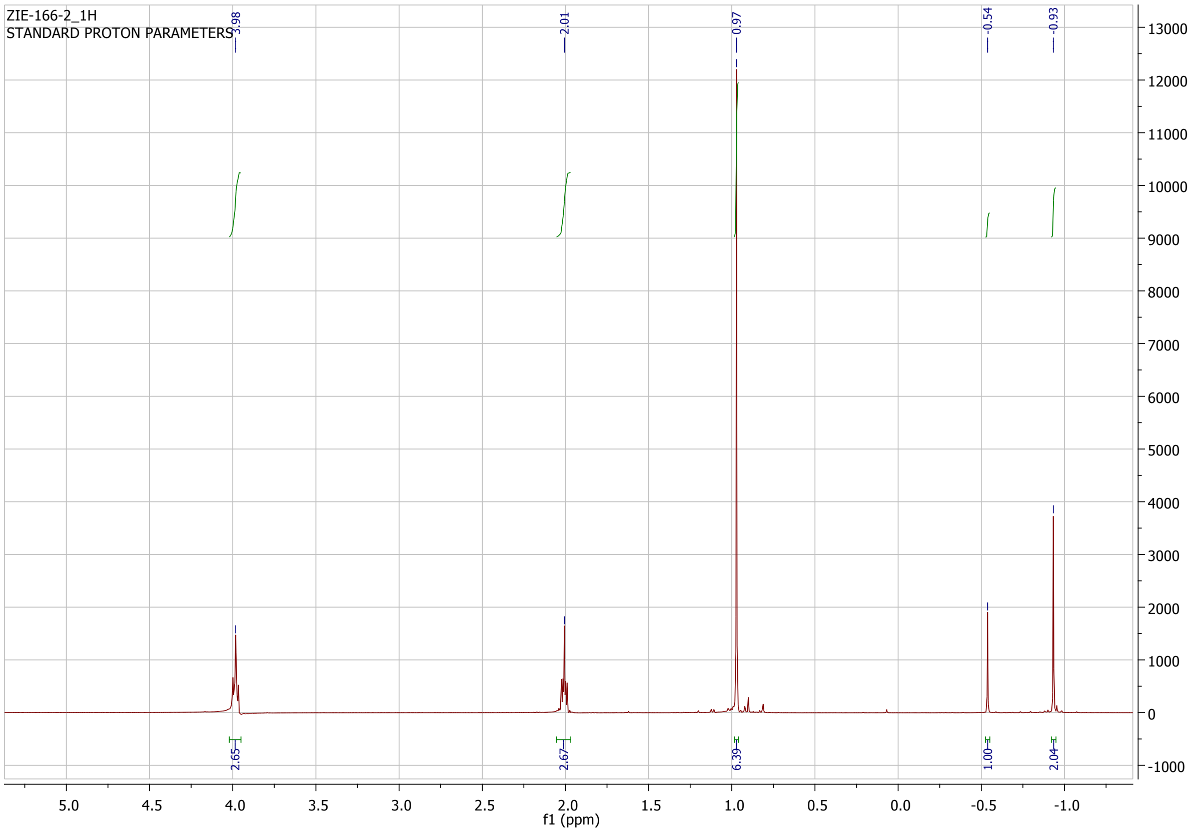

Fig. 11S. ^{1}H NMR spectrum of the distillate obtained from the reaction mixture of the compound **6** with Me_3Al. The spectrum indicates the presence of t-Bu groups bonded to gallium and Me groups bonded to aluminum atoms. δ: 3.98 (m, THF), 2.01 (m, THF), 0.97 (s, t-BuGa), -0.54 (s, MeAl), -0.93 (s, MeAl) ppm.

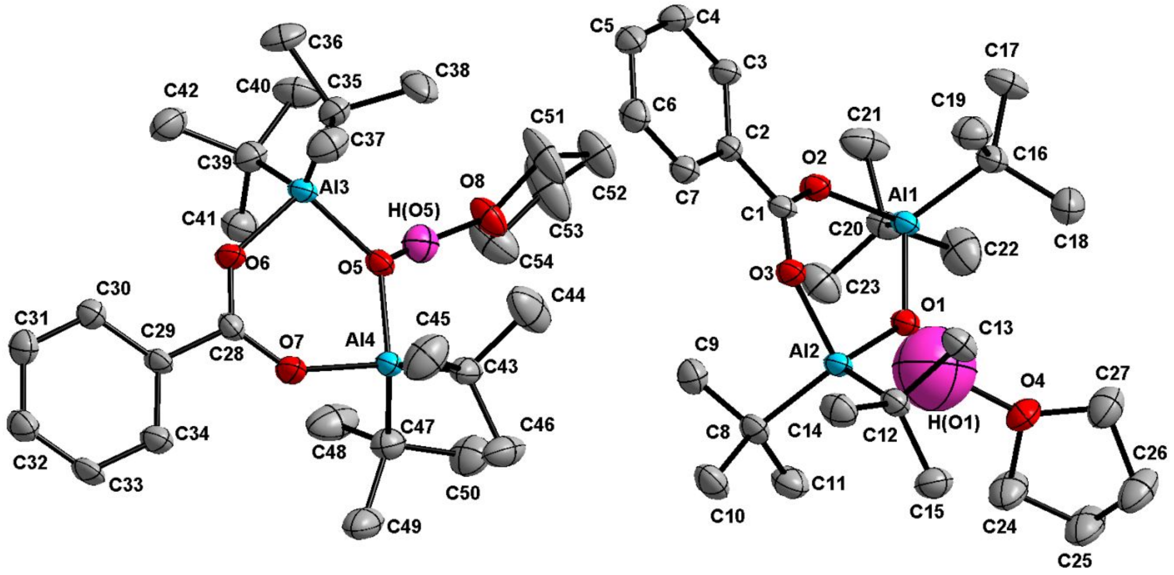

Fig. 12S. Thermal ellipsoid plot (50% probability) of the compound **1**. Hydrogen atoms (besides H(05) and H(01) atoms) have been omitted for the sake of clarity. There are two types of molecules in the unit cell that differ in bond lengths and angle sizes. Selected bonds and distances (Å) and angles (°): Al(1)-O(1) 1.840(4), Al(1)-O(2) 1.841(4), Al(1)-C(20) 1.990(6), Al(1)-C(16) 1.993(6), Al(2)-O(1) 1.838(4), Al(2)-O(3) 1.841(4), Al(2)-C(12) 1.988(6), Al(2)-C(8) 1.992(5), O(1)-H(O1) 0.95(2), Al(3)-O(5) 1.832(4), Al(3)-O(6) 1.846(4), Al(3)-C(35) 1.993(6), Al(3)-C(39) 1.996(6), Al(4)-O(7) 1.836(4), Al(4)-O(5) 1.842(4), Al(4)-C(47) 1.987(6), Al(4)-C(43) 1.987(6), O(5)-H(O5) 0.95(2), O(1)-Al(1)-O(2) 97.6(2), O(1)-Al(2)-O(3) 98.2(2), O(3)-C(1)-O(2) 123.9(4), Al(2)-O(1)-Al(1) 128.1(2), O(5)-Al(3)-O(6) 98.1(2), O(7)-Al(4)-O(5) 96.7(2), Al(3)-O(5)-Al(4) 129.3(2), O(6)-C(28)-O(7) 123.7(4).

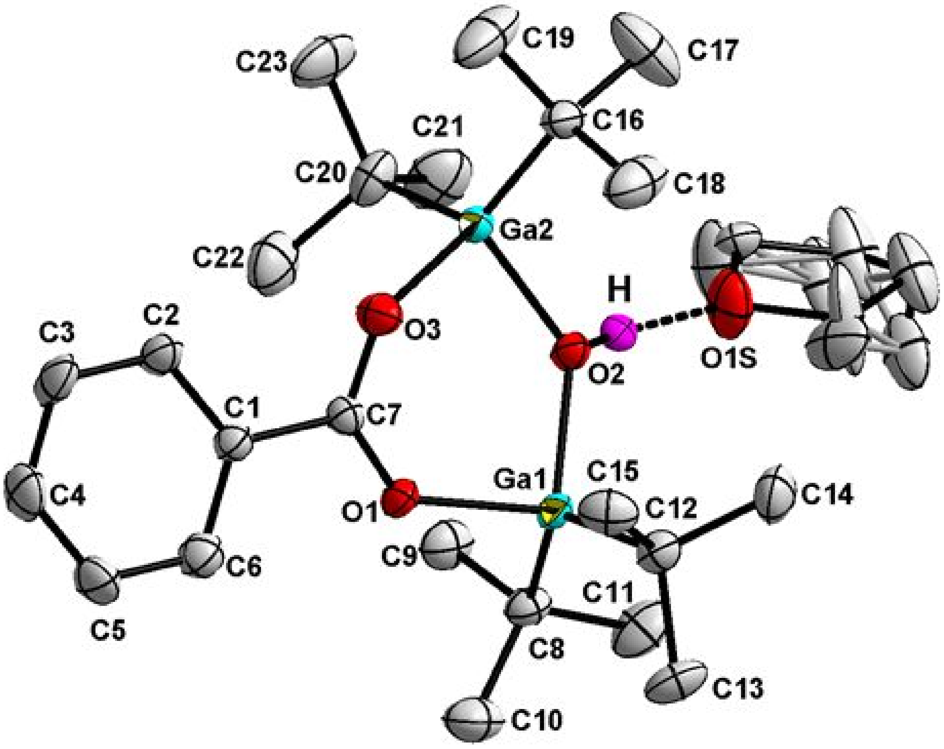

Fig. 13S. Thermal ellipsoid plot (50% probability) of the compound **4**. Hydrogen atoms (besides H20 atom) have been omitted for the sake of clarity. Positions of THF atoms are disordered. Selected bonds and distances (Å) and angles (°): Ga(1)-O(2) 1.929(2), Ga(1)-O(1) 1.967(2), Ga(1)-C(8) 1.999(3), Ga(1)-C(12) 2.000(3), Ga(2)-O(2) 1.933(2), Ga(2)-O(3), 1.968(2), Ga(2)-C(16) 1.988(3), Ga(2)-C(20) 1.990(3), O(1)-C(7) 1.263(3), O(2)-H(2O) 0.88(4), O(3)-C(7) 1.253(3), O(1S)-H(20) 1.850(3), O(2)-Ga(1)-O(1) 96.11(7), O(2)-Ga(2)-O(3) 95.37(7), Ga(1)-O(2)-Ga(2) 128.25(9), Ga(1)-O(2)-H(2O) 118.(2), Ga(2)-O(2)-H(2O) 112(2), C(7)-O(1)-Ga(1) 132.8(2), C(7)-O(3)-Ga(2) 131.9(2), O(2)-H(20)-O(1S) 173.9(3).

Table 1S. Yields of compounds **1-6**.

	Yield of compounds without purification[a] (after crystallization[b]) [%][c]			
	Methods of synthesis			
Compounds	**a**	**b**	**c**	**d**
**1**	95 (45)	91	96	96 (40)
**2**	97 (15)	92	90	92
**3**	90 (40)	95	92	90
**4**	96 (30)	85	89	90
**5**	95 (21)	92	95	90
**6**	96 (25)	90	92	89

[a] Yield of a precipitate after distilling off the solvent and volatiles from the reaction mixture.

[b] Yield of a crystalline solid obtained after crystallization.

[c] Determined based on the amount of acid used in the reaction.

Table 2S. Crystal data and data collection parameters for the compounds **1**, **2**, **4**, **7** and **8**.

	**1**	**2**	**4**	**7**	**8**
Empirical formula	$C_{27}H_{50}Al_2O_4$	$C_{25}H_{53}Al_2O_4$	$C_{27}H_{50}Ga_2O_4$	$C_{29}H_{55}Al_3O_4$	$C_{28}H_{44}Al_4 O_8$
Formula weight	492.63	471.63	578.11	548.67	616.55
Temperature (K)	100(2)	100(2)	130(2)	100(2)	100(2)
Wavelength (Å)	0.71073	0.71073	0.71073	1.54184	1.54184
Crystal system	triclinic	triclinic	monoclinic	monoclinic	monoclinic
Space group	P -1	P -1	P21/c	P 21/c	P 21/n
a(Å)	9.1882(5)	8.640(2)	9.1150(7)	10.5149(1)	14.9168(1)
b(Å)	17.0855(9)	9.889(2)	17.282(1)	13.5725(2)	11.6503(1)
c(Å)	19.6970(12)	10.317(2)	19.856(1)	23.8543(3)	19.2273(2)
α(°)	89.579(2)	67.798(7)	90	90	90
β(°)	80.984(2)	72.536(7)	98.290(2)	101.314(1)	96.300(1)
γ(°)	88.867(2)	72.632(7)	90	90	90
V($Å^3$)	3053.3(3)	761.3(2)	3095.2(4)	3338.17(7)	3321.24(5)
Z	4	1	4	4	4
D_{calc}(g cm^{-3})	1.072	1.029	1.241	1.092	1.233
Absorption coefficient (mm^{-1})	0.121	0.119	1.767	1.260	1.669
F(000)	1080	261	1224	1200	1312
Crystal size (mm)	0.568×0.321×0.226	0.453×0.153×0.088	0.222×0.176×0.152	0.18×0.09×0.04	0.14 x 0.09 x 0.06

Θ range for data collection (°)	2.25 to 25.04	2.18 to 25.04	2.258 to 25.314	3.2520 to 71.4790	3.567 to 70.081
Index ranges	−10 ≤h ≤10, −20 ≤k ≤20, 0 ≤l ≤ 23	−10 ≤h ≤10, −11 ≤k ≤11, −12 ≤l ≤ 12	−10 ≤h ≤10, 0 ≤k ≤20, 0 ≤l ≤ 23	−12 ≤h ≤12, -15 ≤k ≤16, -29 ≤l ≤ 29	−18 ≤h ≤18, -14 ≤k ≤13, -18 ≤l ≤ 23
Reflections collected	10744	25246	119137	16287	39573
Independent reflections	10744 [R_{int} = 0.1191]	2704 [R_{int}= 0.0463]	5672 [R_{int}=0.0530]	6448 [R_{int}= 0.0157]	6299 [R_{int}= 0.0249]
Refinement method	Full–matrix least–squares on F^2	Full-matrix least-squares on F^2	Full-matrix least-squares on F^2	Full-matrix least-squares on F^2	Full-matrix least-squares on F^2
Data / restraints / parameters	10744 / 2 / 627	2704/ 10 / 319	5672/ 10 / 345	6448/ 30/ 364	6299/ 0/ 367
Goodness-of-fit on F^2	1.053	1.088	1.030	0.893	1.029
Final R indices [I>2σ(I)]	R_1 = 0.0998, wR_2 = 0.2901	R_1 = 0.0565, wR_2 = 0.1416	R_1 = 0.0311, wR_2 = 0.0715	R_1 = 0.0440, wR_2 = 0.1161	R_1 = 0.0314, wR_2 = 0.0848
R indices (all data)	R_1 = 0.1101, wR_2 = 0.2988	R_1 = 0.0659, wR_2 = 0.1478	R_1 = 0.0378, wR_2 = 0.0715	R_1 = 0.0465, wR_2 = 0.1179	R_1 = 0.0330, wR_2 = 0.0863
Max/Min of residual electron density	1.264 to -0.545	0.280 to -0.297	0.666 to -0.367	0.644 to - 0.452	0.485 to -0.443
